# Studies of Benzotriazole on and into the Copper Electrodeposited Layer by Cyclic Voltammetry, Time-of-Flight Secondary-Ion Mass Spectrometry, Atomic Force Microscopy, and Surface Enhanced Raman Spectroscopy

**DOI:** 10.3390/molecules28155912

**Published:** 2023-08-06

**Authors:** Robert Mroczka, Agnieszka Słodkowska

**Affiliations:** Laboratory of X-ray Optics, Department of Chemistry, Institute of Biological Sciences, Faculty of Medicine, The John Paul II Catholic University of Lublin, Konstantynów 1J, 20-708 Lublin, Poland; agnieszka.slodkowska@kul.pl

**Keywords:** copper electrodeposition, benzotriazole, cyclic voltammetry, TOF-SIMS, AFM, SERS

## Abstract

Benzotriazole (BTA) is an important compound that demonstrates the strongest anticorrosion properties of copper and plays a role as a leveler and an additive to the electroplating bath for control of the roughness and corrosion resistance of the electrodeposited copper layer. In this paper, we combined cyclic voltammetry (CV), time-of-flight secondary-ion mass spectrometry (TOF-SIMS), surface enhanced Raman spectroscopy (SERS), and atomic force microscopy (AFM) to study the interaction of BTA with copper surfaces at varied concentrations with and without the presence of chloride ions. We identified the most relevant molecular copper and its complex forms with BTA on the copper electrodeposited layer. BTA is adsorbed and incorporated into the copper surface in monomeric, dimeric, trimeric, tetrameric, and pentameric forms, inhibiting the copper electrodeposition. The addition of chloride ions diminishes the inhibiting properties of BTA. The Cu-BTA-Cl complexes were identified in the forms C_12_H_8_N_6_Cu_2_Cl^−^ and C_6_H_4_N_3_CuCl^−^. Coadsorption of chloride ions and BTA molecules depends on their concentration and applied potential. Chloride ions are replaced by BTA molecules. BTA and chloride ions, depending on their concentration and applied potential, control the copper nucleation processes at the micro- and nanoscales. We compared the abilities and limitations of TOF-SIMS and SERS for studies of the interactions of benzotriazole with copper and chloride ions at the molecular level.

## 1. Introduction

Copper electrodeposition plays an important role in the deposition of copper coatings with varied physicochemical properties that can be controlled by the composition of the electroplating bath, e.g., the applied current densities, the time of the deposition process, etc. [1,2,3,4,5,6]. Due to this reason, copper electrodeposited layers find numerous applications in nano- and micro-electronics [7], as well as as a decorative and protective layer [8]. To control the nano- and microstructure of the deposited copper layer, functional organic additives such as Cl^−^ [9,10,11], MPS/SPS [2,12,13,14,15,16], and PEG [17,18,19] are used. Benzotriazole is applied as a leveler that promotes deposition of a smooth copper surface by inhibition of copper adatom diffusion and passivation of growing copper islands by effective adsorption of BTA molecules on the copper surface [20,21,22,23,24]. Moreover, BTA is known as the strongest copper corrosion inhibitor that controls unfavorable reactions that can potentially occur on the surface [25,26]. Levelling and brightening abilities of BTA during copper electrodeposition are connected with the number of nitrogen atoms chemically bonded to the copper that increase the number of nucleated copper with reduced size [27,28,29,30]. Moreover, N-heterocyclic compounds [31] and their oligomers such as 1,3-bis(1-imidazolyl)propane [32], polyethyleneimine or DTAC [33,34,35], Benzyl-Containing Quaternary Ammonium Salt [36], 1-(4-Ethoxyphenyl)-5-mercapto-1H-tetrazole [37], and Janus Green B [38] can be used as levelers for acid copper plating. BTA can also form an inhibiting film on the copper electrodeposited layer after immersion in BTA solution [39]. Numerous studies were devoted to the interaction of BTA with oxide-free [26,40,41,42,43,44,45,46,47,48,49,50,51,52] and oxide-covered [28,52] copper surfaces. However, the exact mechanism of interaction between BTA and copper surfaces is very often controversial and contradictory [25,53,54]. Generally, it is proposed that BTA can interact with copper in two ways: (1) via the formation of copper metal complexes through nitrogen atoms [25,28,30,47,55,56] that develop polymeric chains [25,26,30,50,52,57,58,59,60,61] or (2) by adsorption in molecular form to the copper surface [43,62,63,64]. The formation of Cu(I)BTA complex form in neutral and acidic (sulfuric acid) solutions is favored by a higher anodic potential and higher pH, while molecular form is favored by a negative and lower potential [43,57,63,64,65]. Nevertheless, analysis of the interaction of BTA with copper surfaces with or without the presence of chloride ions under real electrodeposition conditions was carried out only by a few researchers [20,21,22,23,24,66,67]. On the other hand, the latter studies [20,67] were based on only the electrochemical balance technique for determining mass changes during cyclic voltammetry studies. Numerous in situ studies realized via the SERS technique are devoted to solutions consisting of only BTA and sulfuric acid [43,57,63,64,65]. The limitation of the SERS technique is the strong suppression of the Raman signal for samples prepared in solution consisting of a high concentration of copper sulfate during copper electrodeposition, which determines a noticeable variation in the morphology of the substrate during copper electrodeposition. On the other hand, XPS studies of BTA-copper interactions were carried out for copper immersed in solution containing 50 mM CuSO_4_ and 10 mM KCl at pH ranging from 4 to 6 [58]. The XRD studies allow for the determination of the molecular Cu(II)BTA [68], Cu(I)BTA [69], and Cu(I)BTACl complexes [70,71] structures. Nevertheless, it is obvious that the molecular interactions of BTA with copper with and without chloride ions under real copper electrodeposition conditions are significantly different. For the determination of the molecular structure and interactions of BTA with the copper surface, a few attempts were carried out using the TOF-SIMS technique [52,72,73,74,75,76]. Moreover, the interaction of similar compounds with copper was examined by TOF-SIMS [35,39]. However, no trials have been devoted to the analysis of the copper electrodeposition under the static regime of TOF-SIMS that allows for the resolution of the molecular structure of Cu-BTA. Instead of that, dynamic modes of TOF-SIMS that allow determination of the distribution of elements incorporated into copper deposits obtained from electroplating baths consisting of BTA were carried out [66,77]. Recently, we showed [15,16,19] that our optimized method that relies on the withdrawal of wire from the electroplating bath during cyclic voltammetry experiments can be successfully applied to the determination of interactions SPS/MPS/Cl/PEG on the copper surface by the TOF-SIMS method.

In this paper, we expand our studies and for the first time combine CV, TOF-SIMS, SERS, and AFM techniques for investigations of BTA interactions with metallic copper and copper ions with and without chloride ions at varied BTA concentrations in the following aspects: (1) the evaluation of the most relevant form of BTA on the copper surface and its reactions with copper and copper ions under open circuit conditions (OCP) and real copper electrodeposition regime; (2) the examination of the complex and molecular formation of Cu-BTA and Cu-BTA-Cl^−^ on the copper surface under OCP and cyclic voltammetry conditions; (3) the determination of the influence of BTA/Cl^−^ copper interaction on the morphology of obtained copper layers at nanoscale; (4) the explanation and molecular analysis incorporated of BTA and Cu-BTA complexes into copper deposit postulated previously [21]. In our work, we applied, for the first time, static TOF-SIMS that provides direct identification of molecular forms of BTA/Cu/Cl^−^ species on the copper surface. The task set in points (1), (2), and (4) allows to formulate, evaluate, and unify the known most relevant interactions between BTA, copper, copper ions, and/or chloride ions postulated during copper electrodeposition [20,21,22,23,24,66,67], which is important for the preparation and optimization of industrial copper electroplating baths containing BTA. Moreover, the proposed mechanism of interactions between BTA and copper can be useful for a better understanding of known models of copper anticorrosion properties of BTA [28,30,40,41,42,43,44,45,46,47,48,49,50,51,52].

## 2. Results and Discussion

### 2.1. Dip-Coating (DC) Experiments on the Bare Nitinol Wire and Pre-Plated Nitinol Wire—Evaluation of the Chemistry of Benzotriazole with Copper and Chloride Ions under a Non-Electrochemical Condition

**Figure 1:**
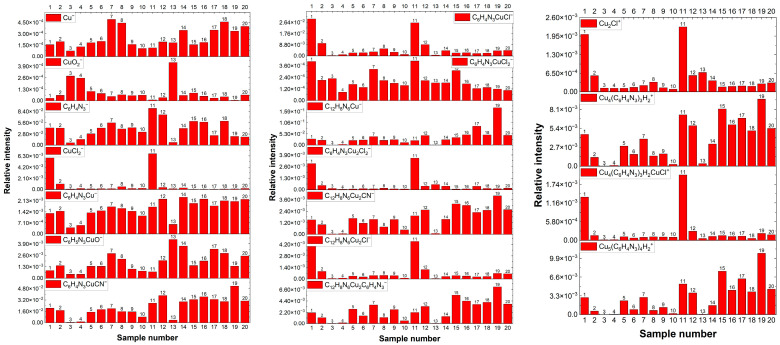
Intensity distribution of selected negative and positive fragments identified in the TOF-SIMS spectra.

Figure 1 shows the distribution of the most prominent negative and positive fragments identified in the TOF-SIMS spectra for samples 1–20. Samples were prepared by immersing nitinol or nitinol wire covered by a copper layer deposited from the base solution into solutions of varied composition (Table 1). The method of preparation is provided in the section Materials and Methods. The *m*/*z* ratios and assignments of selected fragments identified in the TOF-SIMS spectra are included in Table 2.

After mixing CuCl_2_ and BTA (1, 2, 11, and 12 samples), green precipitation was formed, while for solutions consisting of sulfuric acid (samples 3, 4, 13, and 14), the precipitation was not formed. For other solutions, no changes were observed. In the highest mass range, three characteristic positive fragments corresponding to the Cu-BTA adducts were identified. The fragments with mass *m*/*z* = 605.89, 703.80, and 786.87 correspond to Cu_4_(BTA)_3^+^_, Cu_4_(BTA)_3_CuCl^+^, Cu_5_(BTA)_4^+^_, and BTA = C_6_H_4_N_3_, respectively. The positive fragments assigned as Cu_4_(BTA)_3^+^_ and Cu_5_(BTA)_4^+^_ were identified previously by TOF-SIMS on the copper surface immersed for 10 min in 0.01 M BTA by Notoya et al. [76]. It showed that in the acidic (pH 1) and alkaline (pH 13) solutions, the fragments Cu_4_(BTA)_3^+^_ and Cu_5_(BTA)_4^+^_ were not observed. The mass fragments 607.8 and 788.8 with the same assignment as listed in Table 2 were identified by ESI-TOF from the dispersion of copper nanoparticles covered by a BTA layer prepared by the modified Brust-Schiffrin method [78].

**Figure 2:**
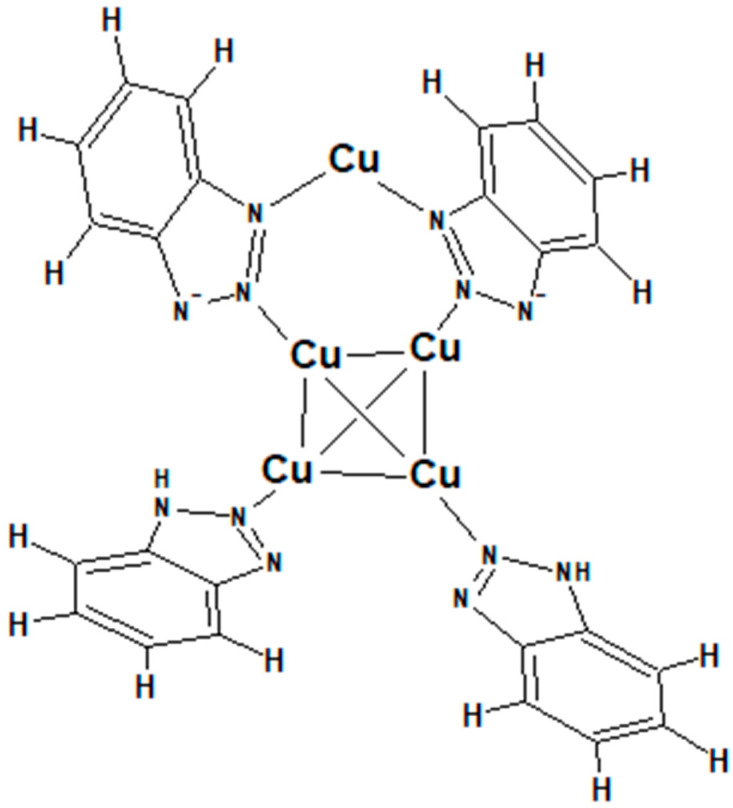
Proposed to simplify the molecular structure of Cu_5_(BTA)_4^+^_. The optimized molecular structure of Cu_6_(BTA)_4_ can be found elsewhere [61].

The authors carried out structural optimization by means of the DFT method and assumed the existence of cluster Cu_6_(BTA)_4_. The cluster Cu_6_(BTA)_4_ consists of a compact tetrahedral Cu_4_ core covered by two BTA-Cu(I)-BTA units. On the other hand, this model is slightly inconsistent with the molecular assignment of Cu_5_(BTA)_4^+^_ identified by mass spectrometry. It seems that the latter fragment identified in TOF-SIMS spectra and in ESI-TOF spectra should consist of a central tetrahedral Cu_4_ core, one BTA-Cu(I)-BTA ligand connected to Cu_4_, and two separated BTAH ligands connected to the central Cu_4_, as shown in Figure 2.

Moreover, the slightly higher yield of Cu_4_(BTA)_3^+^_ than Cu_5_(BTA)_4^+^_ suggests a second, very stable structure containing three BTA ligands connected to a central, compact Cu_4_ core. In this scenario, one BTA ligand forms two N-Cu bonds with core Cu4, while two other BTAH ligands are connected only by one N-Cu bond. The second possible structure of Cu-BTA adducts that explain interactions of BTA with copper assumes the existence of a polymeric linear structure of [Cu(I)-BTA-Cu(I)]_n._ For linear forms, we should expect that the yield of fragments decreases as the mass of fragments increases, which is typical for linear polymers identified in the TOF-SIMS mass spectra [19]. Cu_4_(BTA)_3^+^_ and Cu_5_(BTA)_4^+^_ in the mass spectra should be lower than the fragments Cu_3_(BTA)_2^+^_ and Cu_2_(BTA)^+^. However, the lack of the fragments Cu_3_(BTA)_2^+^_ and Cu_2_(BTA)^+^ with significantly greater abundance than Cu_4_(BTA)_3^+^_ and Cu_5_(BTA)_4^+^_ is determined by the compact, cyclic form of the latter fragments.

Only the fragment Cu_2_(BTA)^+^ with a similar yield was identified, pointing out that BTA can also be bonded to Cu in monodentate form.

The above consideration supports the model of cluster molecular structure proposed in Figure 2 and by Salorinne et al. [78].

For samples 1 and 11, we identified the fragment Cu_4_(BTA)_3_CuCl^+^. After rinsing (samples 2 and 12), the intensity of Cu_4_(BTA)_3_CuCl^+^ significantly diminishes for nitinol on the copper substrate. After rinsing, the intensity of Cu_4_(BTA)_3_CuClH_2^+^_ is very low and similar to the samples that do not contain chloride ions (samples 3–10 and 13–20). It means that the Cu-BTA-Cl complex is very soluble in rinsing solution. In acidic solutions (samples 3, 4, 13, and 14), the Cu-BTA-Cl complex and green precipitation were not observed. Furthermore, the lack of fragment Cu_4_(BTA)_3_CuCl^+^ for samples 3, 13 and 4, 14 is consistent with this observation. For sample 13, the very low intensity of the fragments Cu_4_(C_6_H_4_N_3_)_3^+^_ and Cu_5_(BTA)_4^+^_ indicates that the formation of the polymeric form of Cu(I)-BTA is strongly inhibited in acidic solutions (pH < 1), as previously reported [43,76,79,80]. However, the significantly lower intensity of Cu_4_(BTA)_3^+^_ for the nitinol substrate (sample 3) in comparison to the copper substrate (sample 13) suggests that the formation of some amount of Cu(I)-BTA polymeric complexes is possible on the copper substrate.

After rinsing, the intensity of the fragment Cu_4_(BTA)_3^+^_ only slightly increases for nitinol substrate (sample 4), while on copper (sample 14), it strongly rises. It means that metallic copper forms chemical bonds with BTA in acidic solution, similarly to the complex Cu(I)-BTA that is observed for samples 11, 12, and 14–20. This observation is supported by the similar distribution of the following fragments: C_6_H_4_N_3_Cu^−^, C_6_H_4_N_3_CuCN^−^, C_12_H_8_N_6_Cu^−^, C_12_H_8_N_6_Cu_2_CN^−^, and C_18_H_12_N_9_Cu_2^−^_. Furthermore, the chemical bonds of BTA with the nitinol substrate were not identified in the TOF-SIMS spectra.

Identification of the negative high mass fragments: C_6_H_4_N_3_- (*m*/*z* = 118), C_6_H_4_N^−^, C_12_H_8_N_6_Cu^−^(*m*/*z* = 299), C_18_H_12_N_9_Cu_2^−^_ (*m*/*z* = 480), C_24_H_16_N_12_Cu_3^−^_ (*m*/*z* = 661), as well as Cu_4_(BTA)_3_ and Cu_5_(BTA)_4_ was reported previously [52,76].

The fragmentation of BTA molecules during bombardment by the Bi primary beam is as follows (1):




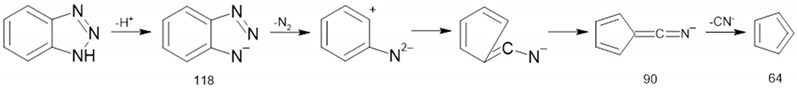

(1)



In the first step, the hydrogen cation H^+^ is released, yielding the anionic form of BTA (*m*/*z* = 118). The free electron pair indicated by the sign minus is highly delocalized on three nitrogen atoms. In the second step, N_2_ is subtracted, giving a carbocation that, after intermolecular arrangement, forms a ketenimine anion (*m*/*z* = 90). Finally, after releasing the cyanide anion CN^−^, the neutral form of 1,2,4-cyclopentatriene is yielded (*m*/*z* = 64). The lack of fragments with mass 64 in positive as well as negative modes proves the proposed pathway of fragmentation of BTA. A similar fragmentation pathway was proposed previously [81]. On the other hand, the experimental mass spectra by electron ionization were obtained in the positive ion mode for masses 119, 91, and 64. In more recent results, BTA characteristic fragments in the negative ion mode were identified for *m*/*z* = 118 after electron spray ionization [78] and by TOF-SIMS [52,76] that strongly support the fragmentation pathway (1). Moreover, for positive ion mode, the fragment with mass 119 was not observed as it corresponds to the neutral form of BTA. On the other hand, we identified C_6_H_6_N_3^+^_ as the protonated form of BTA in the positive ion mode.

The anionic fragments of Cu_x_(BTA)_y_^−^ are yielded from linear dimeric (BTA-Cu-BTA), trimeric (BTA-Cu-BTA), and tetrameric forms (BTA-Cu-BTA-Cu-BTA-Cu) of Cu-BTA. This hypothesis is based on the intensity ratio of the fragments *m*/*z* 299, 480, and 661 that is equal to 662:87:1.7 identified in the TOF-SIMS spectra. Such an intensity distribution is typical for linear forms of polymers, where the intensity of larger mass fragments rapidly decreases. As it was pointed out in the positive mass spectra, we did not notice this kind of fragmentation. In the case of the polymeric form of [Cu-(BTA)]_n_ on the copper surface, every BTA subunit involves one nitrogen bond to the copper substrate, while the remaining two nitrogen atoms are connected to two copper adatoms [28,30]. In consequence, two atoms of nitrogen are coordinated to two copper adatoms, while in dimeric form, only one atom of nitrogen is coordinated to one copper adatom. As a result, the less coordinated Cu atoms involved in the Cu-BTA complex structure, the higher the binding energy of every BTA unit [27,30,82].

The most prominent fragment of BTA in deprotonated form (C_6_H_4_N_3^−^_) was detected in all samples except samples 3 and 13, which contained sulfuric acid. For those samples, the intensity of all negative fragments is significantly reduced, with the exception of the hydroxylated form of BTA, C_6_H_4_N_3_CuOH^−^, for sample 13. After rinsing, a significant increase in all BTA and Cu-BTA fragments for copper substrate (sample 14) occurs, while for nitinol, that effect is not observed (sample 4). We can conclude that the complex forms of Cu(I)-BTA can be formed on the copper surface in acidic solution.

The distribution of the intensity fragments CuCl_2−_, C_6_H_4_N_3_CuCl^−^, C_6_H_4_N_3_Cu_2_Cl_2^−^_, C_12_H_8_N_6_Cu_2_Cl^−^, Cu_2_Cl^+^, and Cu_4_(BTA)_3_CuCl^+^ is very similar. High intensity of the latter fragments is observed for samples 1 and 11 from solutions containing green complex and significantly decreases after rinsing (samples 2 and 12). It proves that the negative fragments containing chloride ions are yielded from the same molecular structure as Cu_4_(BTA)_3_CuCl^+^.

### 2.2. Chemistry of Copper Surface after Copper Electrodeposition from Electrolyte Containing BTA without Chloride Ions—Cyclic Voltammetry and TOF-SIMS Measurements

**Figure 3:**
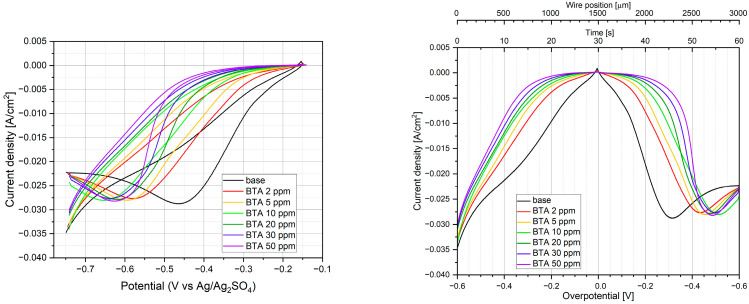
Cyclic voltammetry for base solution and BTA concentrations: 2, 5, 10, 20, 30, and 50 ppm. On the left side, in classical fashion, the right plot shows the wire position, time of electrodeposition, and overpotential. Sweep potential speed: 20 mV/s.

Figure 3 depicts cyclic voltammetry curves for 0, 2, 5, 10, 20, 30, and 50 ppm of BTA in solution containing 0.225 M CuSO_4_ and 0.56 M H_2_SO_4_. For the base electrolyte, during reverse scan, a cathodic peak around −0.25 V (V vs. Ag/Ag_2_SO_4_) occurs (Figure 3). It suggests that copper deposition can be controlled by diffusion. After injection of BTA, the cathodic peak is shifted towards more negative potentials for reverse scanning. The greater the BTA concentration, the greater the shift of potential toward a negative value (Figure 3). On the other hand, for forward scanning, the current density is constantly reduced with increasing BTA concentration. It means that BTA acts as a suppressor and increases overpotential during copper electrodeposition. Similar findings were reported previously [20,21,22].

For all CV curves, significant hysteresis is clearly visible. It may be determined by the changes in surface roughness for the forward and reverse scans. When the coordination number of copper atoms on the surface decreases (surface roughness increases), the adsorption energy of BTA increases [28,82]. Surface roughness and morphology of copper surfaces at the nanoscale were examined by atomic force microscopy (see Section 2.11). The occurrence of hysteresis was also observed during copper electrodeposition in the presence of BTA [22] and was explained by different kinds of substrate during forward scan (gold) and reverse scan (copper).

**Figure 4:**
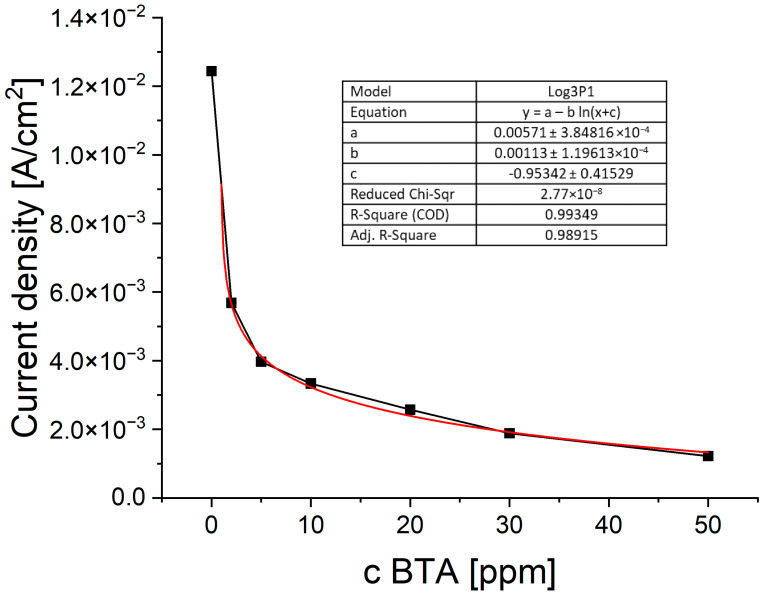
Current density for: 0, 2, 5, 10, 20, 30, and 50 ppm of BTA at wire position 900 µm. It corresponds to the overpotential of −0.25 V for forward scanning (see Figure 3). The red line corresponds to the fitted formula: j_c_ = a − b ln(C_BTA_ + c), where parameters are: a = 0.0057, b = −0.0011, and c = −0.95.

The current density as a function of BTA at wire position 900 µm is shown in Figure 4.

The distribution of current density demonstrates logarithmic behavior according to the formula:

j_c_ = a − b ln(C_BTA_ + c) (2)

where j_c_—cathodic current density; C_BTA_—BTA concentration (ppm); and a, b, c—parameters.

As can be seen in Figure 4, Equation (5) is valid for C_BTA_ ≥ 1 ppm. In a similar way, we fitted data at wire positions 0.3, 0.6, and 1.2 mm, which corresponded to forward scans. Parameter a decreases as a function of wire position since the applied cathodic potential decreases. For C_BTA_ ≥ 5 ppm, the parameter c is negligible, and the decrease in C_BTA_ in the bracket of formula (5) is less than 10%. Parameter b is an important factor that plays a role in the correction coefficient for the whole range of applied overpotentials.

**Figure 5:**
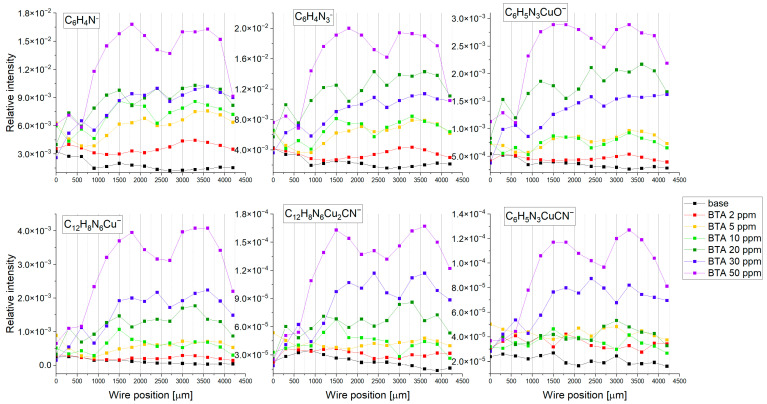
Intensity distribution of the fragments C_6_H_4_N^−^, C_6_H_4_N_3^−^_, C_6_H_4_N_3_CuOH^−^, C_12_H_8_N_6_Cu^−^, C_12_H_8_N_9_Cu_2_CN^−^, and C_6_H_4_N_3_CuOH^−^ as a function of wire position.

Figure 5 depicts the distribution of intensity of the selected fragments: C_6_H_4_N^−^, C_6_H_4_N_3^−^_, C_6_H_4_N_3_CuOH^−^, C_12_H_8_N_6_Cu^−^, and C_18_H_12_N_9_Cu_2^−^_ identified in the negative ion mode and Cu_4_(BTA)_3^+^_, Cu_5_(BTA)_4^+^_ in the positive mode as a function of wire position for the base solution and after addition of BTA in the following concentrations: 0, 2, 5, 10, 20, 30, and 50 ppm.

The position of the wire from 0 to 3000 µm corresponds to the applied potential during the cyclic voltammetry experiment. The wire position from 0 to 1500 µm corresponds to the forward scan, while 1500 to 3000 µm corresponds to the reverse scan. The intensity of C_6_H_4_N^−^ rises with increasing BTA in solution. Only for 30 ppm of BTA is the intensity of C_6_H_4_N^−^ is similar to that for 20 ppm. At 2 ppm of BTA, the intensity is rather stable for the whole CV potential range (wire position 0–3000 µm), with a slight tendency to diminish for lower potentials for forward as well as reverse scan.

The hydroxylated form of BTA assigned to the fragment C_6_H_4_N_3_CuOH^−^ demonstrates roughly similar distribution to the deprotonated form of BTA, C_6_H_4_N_3^−^_.

The fragment C_6_H_4_N_3_CuOH^−^ can be yielded via hydroxylation of the parental BTA molecule and adsorbed via two nitrogen atoms to the copper substrate, while the third nitrogen atom can be attached to the Cu adatom. Under these circumstances, Cu adatom can be hydroxylated during the rinsing of the sample after copper electrodeposition as follows:

C_6_H_4_N_3_(ads) Cu(ad) + OH^−^ → C_6_H_4_N_3_CuOH^−^ (3)

where C_6_H_4_N_3_(ads) stands for the adsorbed BTA molecule and Cu(ad)—the Cu adatom attached to the BTA.

During sputtering in the TOF-SIMS chamber, the fragment C_6_H_4_N_3_CuOH^−^ is released from the copper substrate by the cleavage of two Cu-N bonds that attach BTA to the copper substrate. On the other hand, the fragment C_6_H_4_N_3_CuOH^−^ was also identified for the dip-coated layer of BTA on copper examined in the previous section and by other researchers [52,76] at pH 2 and pH 7. It means that after water evaporation, hydroxylation or oxidation can lead to the same negative ion.

The second important fragment in the negative mode, C_12_H_8_N_6_Cu^−^, corresponds to the dimeric organometallic complex Cu(BTA)_2^−^_. The molecular structure of Cu(BTA)_2^−^_ was studied by the DFT method [25,28,82]. No oxidation or hydroxylated form of Cu(BTA)_2^−^_ was observed. On the other hand, at higher BTA concentrations (20, 30, and 50 ppm), we can expect that BTA exists mostly in dimeric Cu(BTA)_2^−^_, trimeric Cu_2_(BTA)_3^−^_, or polymeric forms. The trimeric form Cu_2_(BTA)_3^−^_, C_18_H_12_N_9_Cu_2^−^_, is yielded from the longer, polymeric chain of BTA. No greater polymeric forms, such as the tetrameric or pentameric forms of BTA, were identified in negative mode. Indeed, the fragment Cu_2_(BTA)_3^−^_ was identified in the TOF-SIMS spectra at 30 and 50 ppm of BTA.

The lack of the dimeric form C_6_H_4_N_3_CuOH^−^ in the TOF-SIMS spectra means that the fragment C_6_H_4_N_3_CuOH^−^ is yielded from single BTA molecules adsorbed on the copper surface (reaction 3) that may coexist with organometallic dimeric forms of BTA.

The contribution of C_12_H_8_N_6_Cu^−^ and Cu_2_(BTA)_3^−^_ is low at 2 and 5 ppm of BTA, respectively. Only at 10 ppm for low overpotentials for forward and reverse scan (wire position 1300–1800 µm), the intensity of the organometallic complex Cu(BTA)_2^−^_ significantly grows. On the other hand, the complex Cu(BTA)_2^−^_ covers the surface for the whole overpotential range of 20, 30, and 50 ppm, respectively. Moreover, the polymeric form Cu_2_(BTA)_3^−^_ is clearly visible at 30 and 50 ppm. The surface coverage of organometallic complexes in dimeric form Cu(BTA)_2^−^_ as well as in polymeric form Cu_2_(BTA)_3^−^_ increases towards more positive potentials, reaching maximum at OCP, while during reverse scan it is rather stable with some tendency to decrease for higher overpotentials (more negative) at 50 ppm (position 2400–3000 µm).

**Figure 6:**
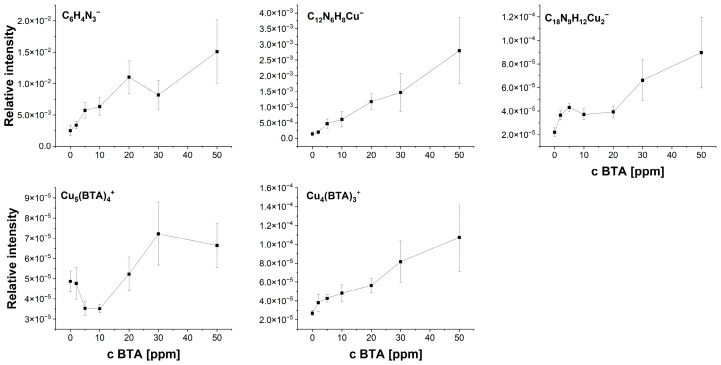
Distribution of intensity of the selected negative fragments: C_12_H_8_N_6_Cu^−^, C_18_H_12_N_9_Cu^−^, C_6_H_4_N_3^−^_, Cu_4_(BTA)_3^+^_, and Cu_5_(BTA)_4^+^_ on the copper surface obtained from solutions contained: 0, 2, 5, 10, 20, 30, and 50 ppm of BTA, and 30 ppm of Cl. The standard deviation corresponds to the variation of intensity along the wire position from 0 to 3.0 mm (see Figure 5).

Figure 6 depicts the relationship between intensity and selected fragments as a function of BTA concentration calculated as a mean value of intensity in the cyclic voltammetry region (wire position 0–3000 µm). The standard deviation is related to the deviations of intensity along the wire position (applied potential). The intensity of C_6_H_4_N^−^ increases up to 20 ppm. Subsequently, at 30 ppm, the intensity of C_6_H_4_N_3^−^_ decreases, which may suggest greater coverage of dimeric, trimeric, tetrameric, and pentameric forms of BTA, while at 50 ppm, the intensity of C_6_H_4_N_3^−^_ rises, reaching its greatest value.

The intensity of the trimeric form Cu_4_(BTA)_3^+^_ logarithmically grows for BTA concentrations from 2 to 20 ppm, while for 30 and 50 ppm, a greater yield is clearly seen.

It may suggest that for 30 and 50 ppm of BTA, most BTA molecules exist in polymeric forms consisting of three BTA ligands connected to one copper atom. On the other hand, Cu_5_(BTA)_4^+^_ is observable only at 30 and 50 ppm of BTA. The latter observation may suggest that the molecular arrangement shown in Figure 2 is favorable only at 30 and 50 ppm, while cluster form Cu_4_(BTA)_3^+^_ is formed for the whole investigated BTA concentration range (2–50 ppm).

The distribution of the anionic trimeric form Cu_2_(BTA)_3^−^_ fluctuates for 2 to 20 ppm of BTA and significantly increases for 30 and 50 ppm. On the other hand, the intensity for the whole BTA concentration is significantly greater than the value observed for the base solution from some impurities. The most linear intensity distribution occurs for the dimeric form C_12_H_8_N_6_Cu^−^. Only at 50 ppm of BTA is greater intensity observed. However, the linear trend for C_12_H_8_N_6_Cu^−^ may slightly vary depending on applied potential (wire position), as indicated by the standard deviation in Figure 6.

It is widely accepted that the mechanism of inhibiting properties of BTA films on the copper surface is related to the formation of adsorbed molecular species of BTA and Cu(I)BTA complexes. The formation of Cu(I)BTA complexes depends on the pH and potential [43,63,64]. The molecular form of BTA dominates for high-acidity solutions (pH < 1) and for greater (more negative) potentials. The steady-state balance [79] is as follows:

Cu^+^ +BTAH ↔ Cu(I)BTA  →−0.34 V (4)

Cu(I)BTA + e^−^ ↔ Cu + BTA-  −0.15 V (5)

However, our TOF-SIMS results show that organometallic complexes in the form C_12_H_6_N_8_Cu^−^ and polymeric forms Cu_2_(BTA)_3^−^_ can be formed under our experimental conditions.

The individual forms of BTA monomeric, dimeric, trimeric, tetrameric, and pentameric depend on the BTA concentration and applied potential (Figure 5 and Figure 6) and result from the interaction of metallic copper and Cu^+^/Cu^2+^ ions. On the other hand, the influence of complex Cu(I)BTA and Cu(II)BTA on the exchange current density cannot be directly examined by TOF-SIMS.

Moreover, it was found that Cu^2+^ can form the Cu(II)(BTA)_4_ complexes at very concentrated solutions of Cu^2+^ (0.1 M) and BTA (0.4 M) at pH 1 [83]. X-ray crystallography studies [68] demonstrated that blue crystals with the formula Cu(SO_4_)(H_2_O)(BTAH)_4_ can be formed in this experiment. When the molar ratio BTA:Cu decreases, two BTA ligands can be replaced by water molecules. We reproduced the latter experiment, and we received blue crystals. After immersion of copper wire into the solution over the solid crystal, rapid precipitation of blue crystals was observed. Under our experimental conditions during the CV experiment, we did not observe any precipitation on the copper surface. However, our electrochemical experimental conditions are significantly different, and we cannot rule out the possibility that an invisible thin solid film in the form of Cu(SO_4_)(H_2_O)(BTAH)_4_ can be formed on the copper surface.

Moreover, it was also proven [83] that similar complexes can be obtained after immersing copper plates in acidic solution in the presence of SO_4_^2−^ ions with the central ion Cu^+^. We reproduced that experiment, and we obtained the yellow deposit on the copper plate after several hours. Both blue crystal (Cu(II)BTA) and yellow deposit (Cu(I)BTA) were insoluble in water and diluted sulfuric acid. We carried out TOF-SIMS and SERS measurements for the yellow deposit obtained on the copper plate, and we identified all negative and positive fragments of BTA that existed on the copper wire prepared under the CV experiment. However, our SERS results (Section 2.6) will show that Raman spectra significantly differ for complexes of Cu(I)BTA and SERS data for copper deposited during the CV experiment.

Overall, the existence of the molecular form of BTA in monomeric form C_6_H_4_N_3_CuOH^−^ and organometallic complexes C_12_H_8_N_6_Cu^−^, Cu_2_(BTA)_3^−^_, Cu_4_(BTA)_3^+^_, and Cu_5_(BTA)_4^+^_ was proven on the copper surface under electrochemical conditions. Molecular adsorption of BTA and complexation of Cu^+^ or Cu^2+^ by BTA play a primary role in inhibiting the properties of BTA under copper electrodeposition. For the revision of the TOF-SIMS results, we carried out SERS measurements (Section 2.6).

### 2.3. Incorporation of BTA into Copper during Copper Electrodeposition from Electrolyte Containing BTA without Chloride Ions—TOF-SIMS Measurements

For a better understanding of the mechanism of copper electrodeposition, we examined the possible incorporation of BTA into copper deposits. The incorporation of benzotriazole was examined by SIMS only in dynamic mode, which does not allow identification of the incorporated molecular form of BTA [66,77]. The copper was electrodeposited for 15 min at a constant current density of −15 mA/cm^2^ from a solution of CuSO_4_ (0.225 M)/H_2_SO_4_ (0.5 M)/BTA (50 ppm). Subsequently, the copper deposit was immersed in 30% nitric acid for 10 s for etching. Vigorous bubbling was observed on the surface after 3 s. After etching, the copper deposit was rinsed with deionized water and dried in the air.

**Figure 7:**
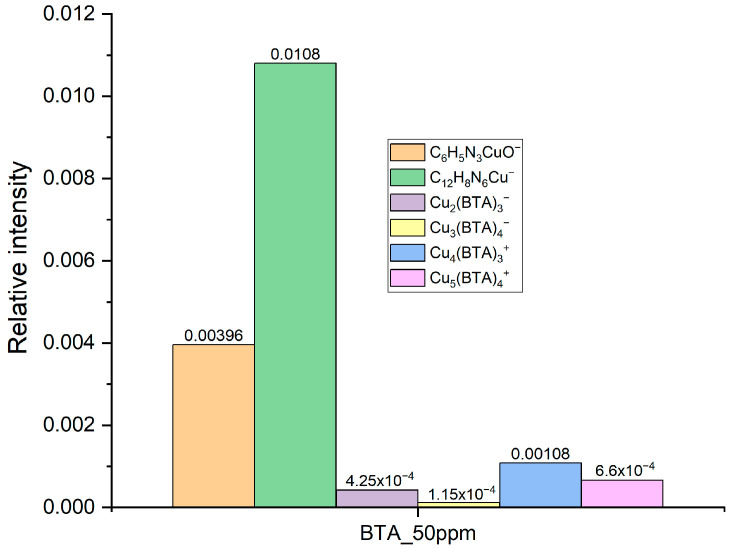
Intensity distribution of C_6_H_4_N_3_CuOH^−^, C_12_H_8_N_6_Cu^−^, Cu_2_(BTA)_3^−^_, Cu_3_(BTA)_4^−^_, Cu_4_(BTA)_3^+^_, and Cu_5_(BTA)_4^+^_ on the copper surface after etching in nitric acid.

The intensity ratio C_12_H_8_N_6_Cu^−^/Cu_4_(BTA)_3^+^_ is about 10, while C_12_H_8_N_6_Cu^−^/C_6_H_4_N_3_CuOH^−^ is about 2.5 (Figure 7). For comparison, the same intensity ratio taken from Figure 2 at wire position 600 µm that corresponds to the current density of −15 mA/cm^2^ (see CV curve, Figure 3) equals 16 and 0.8, respectively. It means that BTA is incorporated into deposits mainly in dimeric C_12_H_8_N_6_Cu^−^, trimeric Cu_4_(BTA)_3^+^_, and tetrameric Cu_5_(BTA)_4^+^_. The yield of the monomeric form C_6_H_4_N_3_CuOH^−^ is significantly reduced (16-fold) in comparison to the dimeric form C_12_H_8_N_6_Cu^−^ identified on the copper surface obtained in the CV experiment. Moreover, the contribution of the incorporated C_12_H_8_N_6_Cu^−^ form in comparison to the polymeric form Cu_4_(BTA)_3^+^_ is also slightly greater.

If we assume that such differences are not determined by different times of electrodeposition (15 min for the etching experiment and 2 min for the CV experiment), we can conclude that incorporation of BTA into the deposit is realized via dimeric forms and polymeric chains. The monomer units are also incorporated, but in a significantly lower amount.

### 2.4. Distribution of BTA and Micromorphology of Copper Surface Obtained from Electroplating Bath without Chloride Ions

**Figure 8:**
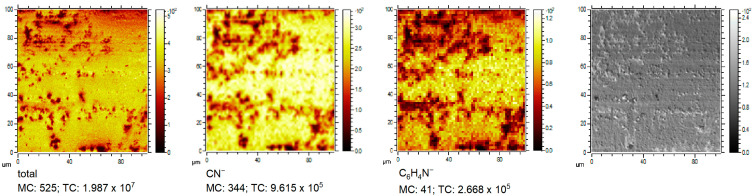
Distribution of selected ions for BTA (50 ppm at wire position 1500 µm). Scanning area: 100 × 100 µm. The right column image shows a backscattered secondary electron image with a resolution of 0.2 µm. The dark areas in the total ion image (left image) correspond to the low amount of BTA as depicted by the CN^−^ and C_6_H_4_N^−^ ions distribution to the crystals visible in the backscattered secondary electron image.

Figure 8 depicts the distribution of the total ion image and selected BTA fragments: CN^−^, C_6_H_4_N_3^−^_, and the backscattered secondary electron image. The darker areas in the total ion image correspond to the low amount of BTA (ions CN^−^ and C_6_H_4_N_3^−^_).

Backscattered secondary electron images (right image) show that crystals are built in areas with very low BTA surface coverage. It directly shows the inhibition properties of BTA in suppressing copper crystal size formation at the microscale. An investigation of the influence of BTA on surface morphology at the nanoscale for all BTA concentrations was examined by AFM (results and discussion in Section 2.11).

### 2.5. Chemistry of Copper Surface after Copper Electrodeposition from Electrolyte Containing BTA without Chloride Ions—SERS Measurements

Surface Enhanced Raman Spectroscopy (SERS) measurements were carried out for the same sample areas as those conducted for TOF-SIMS. According to our best knowledge, combined studies of TOF-SIMS and SERS for copper electrodeposited layers have never been performed. SERS measurements were conducted with 5 µm steps on the wire area of 40 × 3500 µm that contains 6300 measurement points. Good-quality SERS spectra containing characteristic bands for BTA were obtained only for samples electrodeposited from solutions consisting of 20, 30, and 50 ppm of BTA.

**Figure 9:**
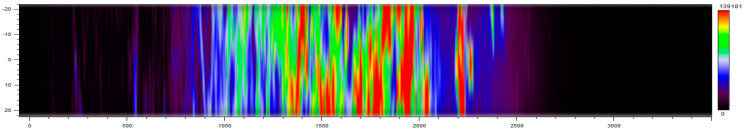
Distribution of intensity in the band 795 cm^−1^ for BTA 30 ppm. The wire position from 0 to 1500 µm corresponds to the forward scan, and the position from 1500 to 3000 µm corresponds to the reverse scan. The position from 3000 to 3500 µm is related to the wire area after switching off current and corresponds to the open circuit potential (OCP).

The distribution of band 795 cm^−1^ in Figure 9 is raw without any postprocessing (smoothing and normalization). It is clearly seen that some spectra recorded at 1500, 1800, and 2200 µm demonstrate greater enhancement than at the other wire position. It is well known that the SERS signal strongly depends on the surface roughness [84,85]. The latter fact determines the significant fluctuation of the Raman signal. The intensity distribution of the band 800 cm^−1^ for BTA 30 ppm is shown in Figure 9.

**Figure 10:**
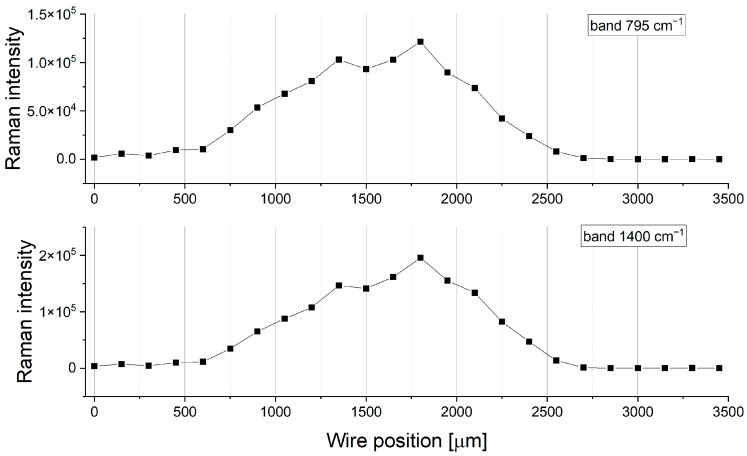
Distribution of Raman intensity bands 795 and 1400 cm^−1^ for 30 ppm of BTA.

The distribution of Raman intensity is not uniform. A significant Raman intensity enhancement around wire position 1800 µm can be observed. On the other hand, the Raman signal diminishes for reverse scans at or wire positions greater than 1800 µm. To receive the average value of Raman intensity for a selected area of wire, the whole sample area was divided into a smaller area of 150 × 80 µm that contains 270 measured points. Subsequently, the average Raman intensity from all 270 measurement points was calculated for selected peaks. The distribution of average Raman intensity for bands 795 cm^−1^ and 1395 cm^−1^ for 30 ppm of BTA is shown in Figure 10.

The adjacent points are separated by 150 µm and correspond to the applied potential with an interval of 60 mV. In this manner, wire position 0 corresponds to the overpotential −0.6 V, position 1000 µm to the overpotential −180 mV for forward scan, 1500 µm to OCP, etc., similarly as it was carried out for TOF-SIMS measurements.

The BTA molecules (bands 793 cm^−1^, 1395 cm^−1^, 1151 cm^−1^, and 1209 cm^−1^) are detected at wire position 750 µm, which corresponds to an overpotential −300 mV. At wire positions from 0 to 750 µm, for overpotentials more negative than −300 mV, the Raman spectra did not contain peaks. When the potential is swept in an anodic direction, the Raman intensity for bands 795 cm^−1^ and 1400 cm^−1^ constantly grows up to −60 mV (wire position 1350 µm). Subsequently, it is stabilized around OCP and again grows up to wire position 1800 µm (overpotential −120 mV, reverse scan). For greater overpotentials, the wire positions continuously decrease up to 2550 µm. At wire positions from 2700 to 3500 µm, no Raman signal was observed. It means that for high overpotentials, more negative than −420 mV BTA molecules are not observed by SERS. Moreover, after switching off current (wire position 3150–3500 µm) under OCP conditions, a Raman signal is also not observed. Contrary to the TOF-SIMS data, at wire positions from 3000 to 4200 µm, BTA molecules were observed with intensity similar to the CV experiment range (wire positions 0–3000 µm).

On the other hand, the CV curves for BTA at 30 ppm (Figure 3) and TOF-SIMS data suggest that BTA surface coverage is still high for greater overpotentials and under OCP after switching off current. It means that the Raman signal is sensitive to the BTA molecules deposited under electrochemical conditions, while under OCP conditions, BTA molecules are not detected, contrary to the TOF-SIMS.

Raman signals can also be significantly diminished for thicker layers of BTA due to the masking of SERS signals from the interface BTA/copper substrate by higher layers of BTA within a multilayer.

For additional insight into this phenomenon, we evaluated SERS data for BTA at 20 ppm and 50 ppm (Figure S1, Supplementary Materials). For 50 ppm of BTA, the Raman signal is strongly enhanced at the wire position from 0 to 600 µm. Subsequently, from 750 to 1800 µm, it is significantly reduced.

It indicates that the SERS signal is limited to the sub- and monolayers of BTA. Consecutively, when BTA layer thickness is reduced at a wire position of 1950 µm, the Raman signal is enhanced due to better access to the copper substrate.

**Figure 11:**
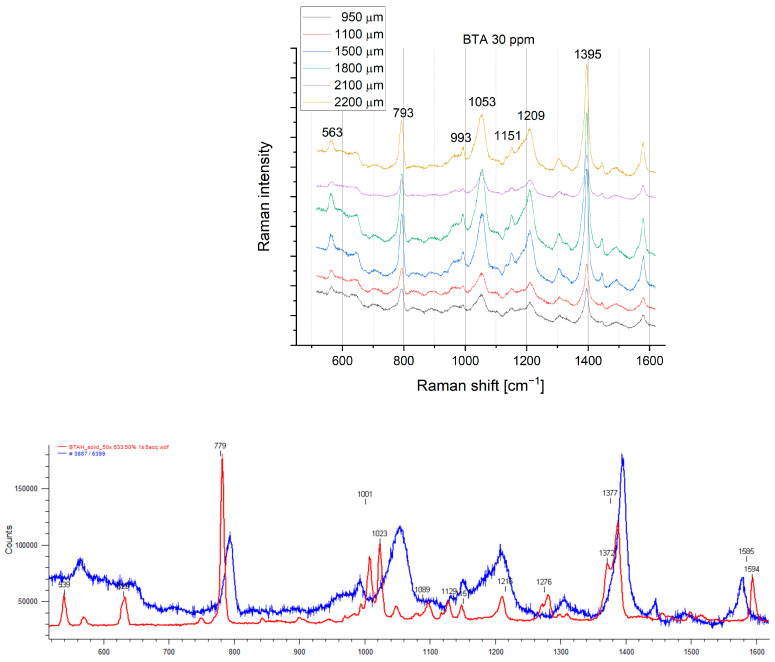
Raman spectra for 30 ppm of BTA at different wire positions (**top graph**) and comparison of Raman spectra for 30 ppm of BTA (blue line) and normal Raman spectra for solid BTAH (red line) (**bottom graph**).

Selected SERS spectra for 30 ppm of BTA at positions of wire: 950, 1100, 1500, 1800, 2100, and 2200 µm at 30 ppm of BTA are shown in Figure 11. For comparison, normal Raman spectra for solid BTA and at 30 ppm of BTA is indicated.

The assignments of selected peaks are included in Table 3.

SERS spectra and normal Raman spectra for BTA demonstrate many similarities, while some differences can be easily noticed (Figure 11). The most prominent band at 782 cm^−1^ for normal Raman for BTAH is blueshifted to 794 cm^−1^ for SERS with reduced intensity.

The lack of Raman shift in bands 793 cm^−1^ and 1395 cm^−1^ for different wire positions (different applied potential) means that the benzene ring does not interact directly with the copper substrate. It indicates that the BTA molecules are oriented perpendicular to the copper surface. This observation is consistent with previous studies of BTA adsorption on the copper surface in an acidic environment [64]. However, there were no SERS studies of BTA molecular orientation during copper electrodeposition under real electrochemical conditions.

On the contrary, the second prominent band (1394 cm^−1^) is only slightly shifted, and intensity significantly increases. The band at 1599 cm^−1^ is redshifted to 1579 cm^−1^. The main structural affection is observed within 1000–1300 cm^−1^. The band 1008 cm^−1^ is redshifted to 990 cm^−1^. The band 1023 cm^−1^ is strongly blueshifted and broadened to 1054 cm^−1^. The latter phenomenon was not observed by Chant and Weaver [64] for in situ measurements of BTAH on copper at pH 2, while after emerging into air, the band 1020 cm^−1^ was blueshifted to 1035 cm^−1^ due to possible oxidation of Cu.

4Cu + 4BTAH(ads) + O_2_ → 4 Cu(I)BTA + 2H_2_O (6)

A similar blueshift of band 1020 cm^−1^ to 1035 cm^−1^ is observed for the Cu(II)(BTA)_4_ blue complex (Figure S2, Supplementary Materials). Considering the above, we can safely assume that a broadened band at 1051 cm^−1^ can arise from the oxidation of metallic copper to Cu^2+^ or Cu^+^. However, the lack of a strong band at 1130 cm^−1^ in SERS spectra that is observed for blue crystal complexes indicates that BTA complexes on the copper surface demonstrate different molecular structures.

Furthermore, the bands 1047 cm^−1^ and 1097 cm^−1^ disappeared, while a significant envelope for the Raman shift from 1150 cm^−1^ to 1210 cm^−1^ is clearly seen. Moreover, the bands 1150 cm^−1^ and 1208 cm^−1^ are still present. The latter band, at 1208 cm^−1^, possesses the shoulder around 1190 cm^−1^. It was found [64] that the disappearance of 1095, 1128, and 1208 cm^−1^ bands is SERS and occurs at pH 2. The latter group of peaks is transformed into an intense broad “envelope” containing partially resolved bands at 1140, 1160, and 1190 cm^−1^ with the assignment shown in Table 3 for Cu(poly). On the other hand, it was observed [64] that in a very acidic environment (pH 0) at electrode potential −0.7 V (SCE), the peak at 1190 cm^−1^ disappeared and two distinct bands at 1150 cm^−1^ and 1125 cm^−1^ instead of 1140, 1160, and 1190 cm^−1^ were noticed. At pH 2, after emerging to air, significant dominance of the band 1190 cm^−1^ over 1140 and 1160 and the blueshift 1020 to 1035 cm^−1^ were considered proof of copper oxidation and spontaneous Cu(I)BTA film formation. The band 1190 cm^−1^ was previously [43,62,63,64] assigned as a Cu(I)BTA complex that is formed at a broad pH and potential range. At pH 0, the band 1190 cm^−1^ [43] constantly rises for potentials more positive than −0.3 V. On the other hand, Honesty et al. [63] calculated the ratio 1190/1140 to estimate the contribution of the Cu(I)BTA complex and molecular form of BTA film to the copper in 0.1 M sulfuric acid. It was concluded that a complex film is formed for a higher positive potential than −0.3 V (vs. Ag/AgCl) for BTA film on roughened polycrystalline copper.

Overall, in our SERS data (Figure 10 and Figure 11), bands 1210 cm^−1^ and 1150 cm^−1^ are clearly seen, while the band around 1190 cm^−1^ is not observed. On the other hand, the significant shoulder at band 1210 cm^−1^ and the broadened strong band at 1051 cm^−1^ indicate the oxidation state of copper and the existence of Cu(I)BTA or Cu(II)BTA complexes. A simultaneously significant amount of BTA in molecular form coexists on the copper surface.

**Figure 12:**
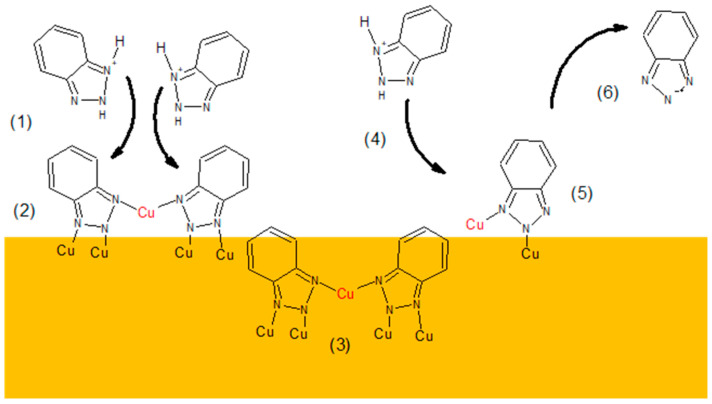
Proposed the cycle of BTA under the copper electrodeposition regime. The red color Cu stands for adatom. The orange color symbolizes the Cu layer. Notice description of brackets in the text.

Taking into account the combined CV, TOF-SIMS, and SERS data, we can propose the following mechanism of interaction of BTA with copper during copper electrodeposition (Figure 12).

In the first step, the protonated form of BTAH_2^+^_ (1) dissociatively adsorbs on the copper substrate in its molecular form.

BTAH_2^+^_ → BTA^•^ + H^+^ + H^+•^ (7)

BTA^•^ + Cu(ad) → Cu (BTA) (8)

Due to the polycrystalline copper substrate, BTAH_2^+^_ molecules can fast adsorb on the substrate in monomeric, dimeric, trimeric, tetrameric, and pentameric forms, depending on the BTA concentration in solution.

The complex in dimeric form, C_12_H_8_N_6_Cu^−^, identified by TOF-SIMS is proceeding in solution as follows:

2 BTAH_2^+^_ + Cu+ → (BTA-Cu-BTA) + 4H^+^ (9)

However, under acidic conditions, after dissociative adsorption, two BTA^•^ radical ions can catch Cu^+^ or Cu_2^+^_ ions, as shown in Figure 12.

2 BTA^•^ + Cu^+^ → BTA-Cu-BTA (10)

For simplicity, in Figure 12, only the most stable dimeric form is shown (2). The dimeric form is bonded to the copper substrate via four Cu-N bonds. Moreover, copper adatom (red color) links two remaining nitrogen atoms. Copper adatoms may be taken from solution in the form of Cu^+^ or Cu^2+^ by BTA molecules to enhance the total binding energy [70]. Results provided in Section 2.2 (incorporation) show that dimeric form is more stable than polymeric form since incorporation of dimeric into copper deposits dominates over polymeric form. On the contrary, on the copper surface, the contribution of polymeric form is slightly greater than that of deposit. The greater binding energy for dimeric rather than polymeric forms was received by Chen et al. [86], while Pejhlan et al. [87] obtained the reverse results.

At 50 ppm, benzotriazole is incorporated into the deposit mostly in the dimeric, trimeric, and tetrameric forms. Only the incorporated dimeric form, which is the most dominant, is shown (3). The monomeric form is also incorporated, though in a significantly lower amount than the other forms. It is due to the lower number of nitrogen atoms involved in bonding a single BTA molecule than longer chains of BTA that desorption is favored in comparison to the dimeric and longer forms. After dissociative adsorption (4) and (5) in the (6) step, the radical form of the BTA species is desorbed. The radical form of BTA can attach hydrogen ions and is released into solution in the protonated form. Consecutively, the protonated form of BTA can undergo pathways (1)–(3) or (4)–(6).

### 2.6. Copper Electrodeposition from Electrolytes Containing BTA with Chloride Ions—Cyclic Voltammetry Studies

Chloride ions are widely used in copper electrodeposition processes as carriers for other additives such as PEG and SPS [13,16,88]. Moreover, in contrast to BTA, Cl ions initiate copper corrosion. Due to this reason, in this paragraph, we evaluated the interaction of BTA and Cl under electrodeposition conditions.

**Figure 13:**
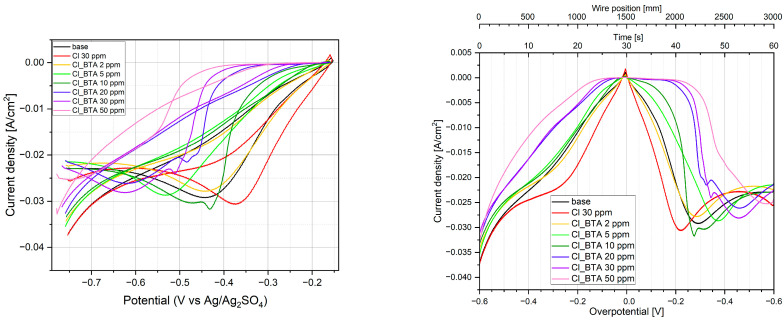
Cyclic voltammetry for base solution and BTA concentrations: 2, 5, 10, 20, 30, and 50 ppm in the presence of chloride ions (30 ppm). The left image demonstrates CV curves in a classical manner; the right plot shows the wire position, time of electrodeposition, and overpotential. Sweep potential speed: 20 mV/s.

Figure 13 shows CV curves for base electrolytes (0.225 M CuSO_4_ and 0.56 M H_2_SO_4_) after addition of 30 ppm Cl and BTA concentrations 2, 5, 10, 20, 30, and 50 ppm in a classical way (left graph) and as a function of wire position (right graph).

The latter plot will be helpful in a direct comparison of the current density at the selected wire position with the relative intensity (surface coverage) of individual components characteristic of BTA and Cl.

It is clearly visible that after the addition of chloride ions, current density increases since Cl^−^ acts as an accelerator [9,10,11]. For a forward scan, the cathodic current peak increases and is shifted to a more positive value. Similar behavior was observed recently [20]. Acceleration behavior is determined by the fact that faster reduction of Cu^2+^ to CuCl forms in insoluble form on the copper surface than reduction of Cu^2+^ to Cu^+^ without the presence of Cl^−^ [89].

After the addition of BTA, CV curves are gradually shifted to lower current densities. For reverse scanning, the cathodic current peak is shifted towards a more negative value that demonstrates the inhibiting properties of BTA. The accelerating abilities of Cl^−^ are gradually reduced. After the addition of 2 ppm of BTA, the accelerating effect of Cl^−^ is completely cancelled, and the CV curve is very similar to that observed for the base solution. A greater amount of BTA enhances the inhibiting properties of BTA. It is interesting that at 10 ppm of BTA, the cathodic peak is greater than for the base solution. Similar behavior was observed elsewhere [20] for BTA (12 ppm) and Cl (10 ppm), a lower CuSO_4_ amount (0.1 M), and a greater concentration of H_2_SO_4_ (1.4 M). In our case, at 10 ppm of BTA, greater inhibition properties for reverse scanning are observed for lower overpotentials (more positive than −0.2 V), while the cathodic peak is greater and shifted towards lower overpotentials. It may suggest that some instability or a lower amount of the BTA-Cu-Cl complex can be formed. Indeed, TOF-SIMS data (Section 2.7) shows that the C_6_H_4_N_3_CuCl^−^ fragment demonstrates lower intensity for 10 ppm of BTA than for 5 ppm. The CV curve demonstrates significant hysteresis, similar to what was reported previously [20]. During the reverse scan, the inhibiting properties of BTA substantially increase in comparison to the forward scan. In our electroplating condition, it is very likely determined by different surface roughness during forward and reverse scans that stimulate the strength of BTA adsorption. In the previous studies [20], it was explained by the fact that the substrate was different (gold) during the forward scan than the reverse scan (copper).

When the coordination number of copper atoms on the substrate decreases, the adsorption energy of BTA increases [28,87]. Morphology at the nanoscale was examined by atomic force microscopy (see Section 2.11).

**Figure 14:**
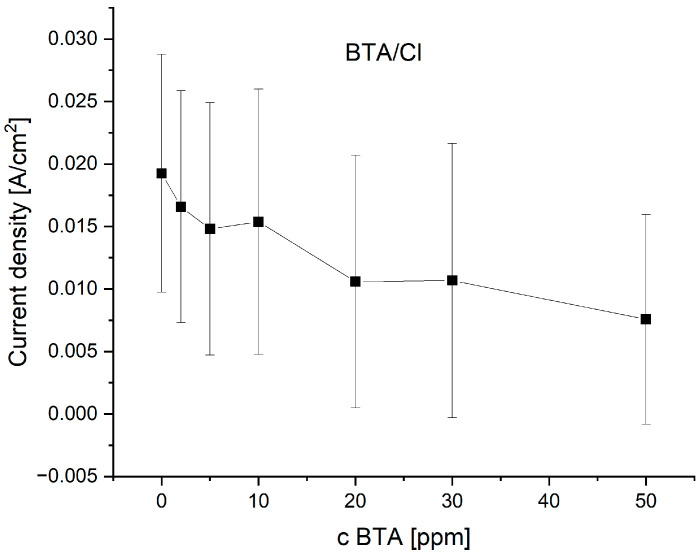
Mean current density as a function of BTA concentration in the presence of chloride ions (30 ppm). Standard deviation stands for current density deviation during the CV experiment for the potential range from −0.6 V to OCP (see Figure 13).

As it is clearly seen in Figure 14, the mean current density fluctuates as a function of BTA concentration. At 2 and 5 ppm of BTA, inhibiting properties of BTA occur. At 10 ppm of BTA, the mean current slightly increases, while at 20 and 30 ppm, it decreases, demonstrating similar values. On the other hand, careful inspection of the CV curve (Figure 13, left plot) shows that inhibiting properties are greater for 30 ppm of BTA than 20 ppm for the whole forward scan and for the reverse scan for potentials more positive than −0.45 V (V vs. Ag/Ag_2_SO_4_). At the highest BTA concentration (50 ppm), inhibiting properties are the strongest. Such behavior may be determined by possible Cu-BTA-Cl complex formation that moderates the inhibiting abilities of BTA in comparison to the molecular and complex forms of BTA identified for BTA without chloride ions. The molecular structure of BTA species in the presence of chloride ions on the copper surface was examined by TOF-SIMS (Section 2.7), SERS (Section 2.10), and AFM (Section 2.11).

### 2.7. Chemistry of Copper Surface Deposited from Electrolytes Containing BTA with Chloride Ions—TOF-SIMS Studies

**Figure 15:**
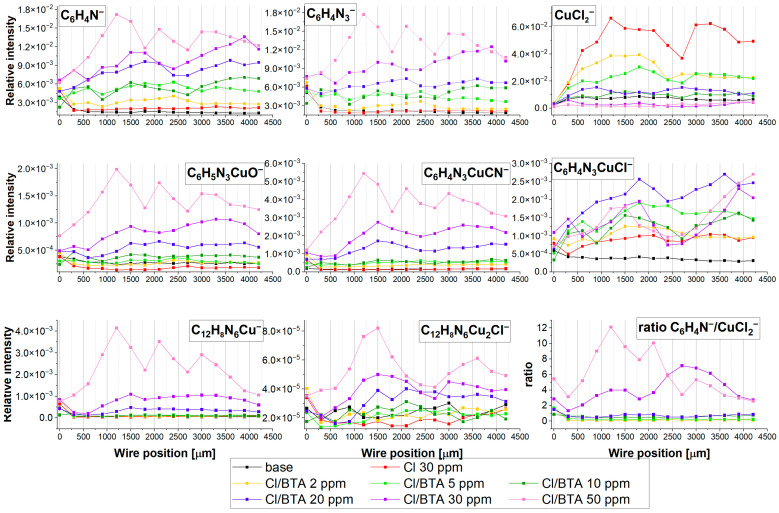
Intensity distribution of the fragments C_6_H_4_N^−^, C_6_H_4_N_3^−^_, C_6_H_4_N_3_CuOH^−^, C_12_H_8_N_6_Cu^−^, C_12_H_8_N_9_Cu_2_CN^−^, and C_6_H_4_N_3_CuOH^−^ and the intensity ratio C_6_H_4_N^−^/CuCl_2^−^_ as a function of wire position.

Figure 15 depicts the intensity distribution of selected negative fragments as a function of wire position (applied potential) for the base electrolyte after addition of 30 ppm Cl and BTA at concentrations 2, 5, 10, 20, 30, and 50 ppm.

The fragment CuCl_2^−^_ stands for chloride ions directly adsorbed on the copper surface. It is clearly seen that its relative intensity decreases as a function of BTA concentration, meaning chloride ions are partially replaced by BTA molecules. The most rapid replacement is observed at 2, 5, and 10 ppm of BTA. Subsequently, increases in CuCl_2^−^_ intensity at 20 ppm of BTA are observed, while at 30 and 50 ppm of BTA, chloride surface coverage is very low, below intensity for the base electrolyte. It may suggest that at 20 ppm of BTA, the greater intensity of CuCl_2^−^_ may be determined by an additional contribution from other chloride compounds such as C_6_H_4_N_3_CuCl or C_12_H_8_N_6_Cu_2_Cl^−^. In this scenario, for 30 and 50 ppm of BTA, chloride ions are not bonded directly to the copper surface since the copper is completely covered by BTA molecules. The small abundance of CuCl_2^−^_ in the base electrolyte is determined by the adsorption of chloride impurities during the transfer of the sample to the TOF-SIMS instrument. That phenomenon was observed and discussed in detail previously [15,16].

**Figure 16:**
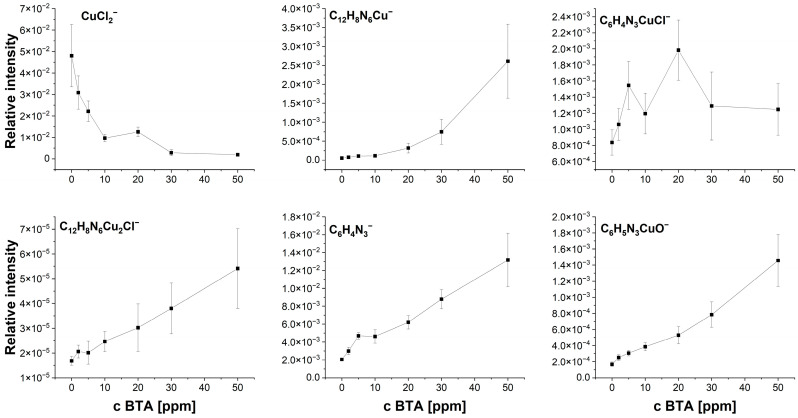
Distribution of intensity of the selected negative fragments: CuCl_2^−^_, C_12_H_8_N_6_Cu^−^, C_6_H_4_N_3_CuCl^−^, C_12_H_8_N_6_Cu_2_Cl^−^, C_6_H_4_N_3^−^_, and C_6_H_4_N_3_CuOH^−^ on the copper surface obtained from solutions contained: 0, 2, 5, 10, 20, 30, and 50 ppm of BTA, and 30 ppm of Cl.

BTA molecules are identified in the following forms: C_12_H_8_N_6_Cu^−^, C_6_H_4_N_3_CuCl^−^, C_12_H_8_N_6_Cu_2_Cl^−^, C_6_H_4_N_3^−^_, and C_6_H_4_N_3_CuOH^−^. Moreover, additional characteristic fragments for BTA were identified (Figure 15). The intensity of C_6_H_4_N_3^−^_ rises at 2 and 5 ppm. Subsequently, at 10 ppm, its amount is comparable to 5 ppm, while for greater BTA concentrations, it linearly grows (Figure 16).

**Figure 17:**
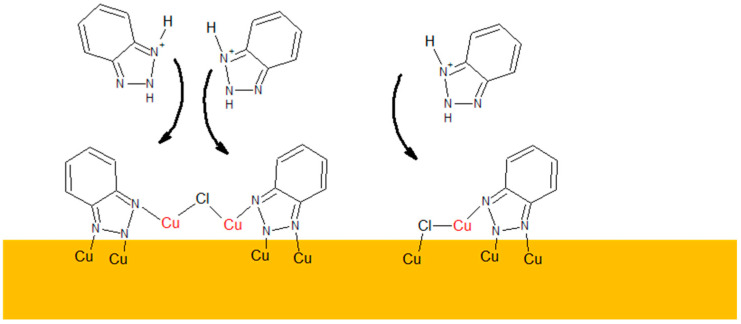
Proposed the cycle of BTA under the copper electrodeposition regime in the presence of chloride ions. The red color Cu stands for adatom. The orange color symbolizes the Cu layer.

However, the fragment C_6_H_4_N_3^−^_ can be yielded from different parental forms of BTA, which makes the identification of the parental form on the copper surface more difficult. More informative fragments having a greater *m*/*z* value, such as C_6_H_4_N_3_CuOH^−^, stand for monomeric BTA in hydroxylated form that grows logarithmically as a function of BTA concentration from 2 to 20 ppm, while at greater BTA concentrations (30 and 50 ppm), they give rise in a more significant way. The intensity of the dimeric form, i.e., C_12_H_8_N_6_Cu^−^, is very low at BTA concentrations of 2, 5, and 10 ppm, while at 20, 30, and 50 ppm, it exponentially grows. It is the opposite situation that occurred in solution without chloride, in which the intensity of the dimeric form significantly grows at 5 and 10 ppm of BTA. It seems that chloride ions covering the copper surface inhibit the formation of BTA in its dimeric form. After the replacement of a significant amount of chloride ions directly bonded to the copper surface at 30 and 50 ppm by BTA molecules, the number of dimeric forms represented by the C_12_H_8_N_6_Cu^−^ fragment constantly grows. The possible Cu-BTA-Cl complex formation can be examined by the distribution of C_6_H_4_N_3_CuCl^−^ and C_12_H_8_N_6_Cu_2_Cl^−^. A very high intensity of these fragments was observed for samples 1 and 11 in the dip-coating experiment (Section 2.1). The abundance of C_12_H_8_N_6_Cu_2_Cl^−^ constantly grows, similarly to that of C_6_H_4_N_3_CuOH^−^. However, at 50 ppm of BTA intensity, the distribution of C_12_H_8_N_6_Cu_2_Cl^−^ is slightly different than other Cu-BTA complexes and demonstrates the highest yield around OCP (wire position 1.5 mm). It strongly suggests that the C_12_H_8_N_6_Cu_2_Cl^−^ complex formation may be realized at greater surface coverage by chloride ions (2, 5, and 10 ppm of BTA) and at a lower amount of Cl directly bonded to the copper surface (30 and 50 ppm of BTA), as shown in Figure 17.

The intensification of non-chloride C_12_H_8_N_6_Cu complex formation in solutions containing greater amounts of BTA (30 and 50 ppm BTA) but without chloride ions is clearly seen. In consequence, the coexistence of C_12_H_8_N_6_Cu_2_Cl^−^ and C_12_H_8_N_6_Cu^−^ complexes at 30 and 50 ppm of BTA occurs.

The complex C_12_H_8_N_6_Cu_2_Cl^−^ formation at 30 and 50 ppm of BTA can be followed via the following pathway:

2CuCl_2_^− +^ 2 C_6_H_6_N_3^+^_ → C_12_H_8_N_6_Cu_2_Cl^−^ + 3Cl^−^ + 4H^+^ (11)

On the other hand, at lower amounts of BTA (2, 5, 10, and 20 ppm), the complex can be formed by the reaction of BTA molecules and chloride ions adsorbed on the copper as follows:

2 C_6_H_4_N_3_^•^(ads) + 2Cu(ad) + Cl^−^ (ad) → C_12_H_8_N_6_Cu_2_Cl^−^ (12)

The formation of complex [Cu(I)ClBTAH]_4_ was postulated by Rubim et al. [65] based on their SERS studies. However, as it was mentioned earlier, SERS does not provide direct identification of the molecular form of BTA-Cu-Cl complexes. Moreover, polymeric complexes in the form of Cu-BTA-Cl studied by XPS were postulated [90].

At greater amounts of BTA (30 and 50 ppm), dissociative adsorption of BTA in deprotonated form to the copper surface is more favorable than adsorption of chloride ions. On the other hand, the adsorption of chloride ions in the complexed form C_12_H_8_N_6_Cu_2_Cl^−^ created in Reaction (1) simultaneously occurs. It arises from the fact that the protonated form of BTA is less hydrated than chloride ions.

The simplified pathway of reactions (11) and (12) is shown in Figure 17.

Different forms of Cu-BTA-Cl complexes may be assigned to C_6_H_4_N_3_CuCl^−^, whose intensity rises to 20 ppm of BTA (at 10 ppm, we observe its lower amount), while at 30 and 50 ppm, BTA significantly decreases. The distribution of C_6_H_4_N_3_CuCl^−^ in comparison to CuCl_2^−^_ demonstrates a reasonable correlation.

At 2 and 5 ppm, the ratio of C_6_H_4_N^−^/CuCl_2^−^_ (Figure 15) is below 0.5 and is constant at wire position from 0 to 2100 µm, while the amount of C_6_H_4_N_3_CuCl^−^ constantly grows. For 10 ppm of BTA, the intensity of C_6_H_4_N_3_CuCl^−^ is significantly reduced due to the greater ratio C_6_H_4_N^−^/CuCl_2^−^_. Subsequently, at 20 ppm of BTA, the C_6_H_4_N^−^/CuCl_2^−^_ ratio gives rise at wire positions 1300 to 2300 µm while the amount of CuCl_2^−^_ simultaneously grows. It means that a greater amount of BTA molecules and chloride ions at this BTA concentration is favorable for Cu-BTA-Cl complex formation. At the greatest BTA concentrations (30 and 50 ppm), the C_6_H_4_N^−^/CuCl_2^−^_ ratio significantly grows, which determines the low amount of possible Cu-BTA-Cl complexes, as proven by the lower intensity of C_6_H_4_N_3_CuCl^−^. On the other hand, it is difficult to conclude that the fragment C_6_H_4_N_3_CuCl^−^ may exist in complex, parental form on the copper surface or be yielded via the reaction of copper sputtered ions during Bi primary beam bombardment. The replacement of chloride ions by BTA shown in Figure 15 is determined by the fact that chloride ions are smaller than BTA and solvate significantly stronger in water solutions than BTA^−^ [87]. In consequence, chloride ions lose competition in adsorption with BTA.

The C_6_H_4_N_3_CuCl^−^ complex formation is realized via the reaction of the protonated form of BTA with the CuCl_2^−^_ chloride complex.

CuCl_2_^− +^ + C_6_H_6_N_3^+^_ → C_6_H_4_N_3_CuCl^−^ + Cl^−^ + 2H^+^ (13)

Or alternatively, after adsorption of BTA on the copper, a chloride ion can be attached to the copper adatom.

C_6_H_4_N_3_Cu (ads) + Cl^−^ → C_6_H_4_N_3_CuCl^−^ (14)

Polymeric forms of Cu-BTA-Cl complexes were postulated previously for benzotriazole [90]. Furthermore, polymeric chloride complexes for 2-mercaptobenzimidazole (MBI) were also reported [91]. On the other hand, the occurrence of cuprous intermediates Cu^+^/Cu(I)BTA/CuCl that partially eliminate the inhibition properties of BTA was recently postulated [20]. TOF-SIMS results show that chloride ions are connected to the BTA molecules through Cu^+^ ions from the solution side and are not directly bonded to the copper substrate, as shown in Figure 17. It is supported by the very low intensity of CuCl_2^−^_, which stands for chloride ions in the form of CuCl, on the copper surface.

To reveal the formation of Cu-BTA-Cl complexes, we carried out SERS measurements (Section 2.10). The SERS technique can provide additional understanding about the mechanism of Cu-BTA-Cl complex formation.

**Figure 18:**
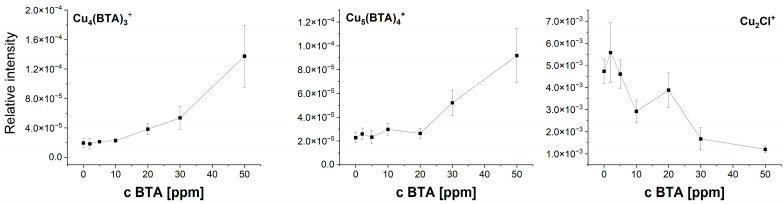
Intensity distribution of Cu_4_(BTA)_3^+^_, Cu_5_(BTA)_4^+^_, and Cu_2_Cl^+^ on the copper surface obtained from solutions contained: 0, 2, 5, 10, 20, 30, and 50 ppm of BTA, and 30 ppm of Cl^−^.

The distribution of selected positive fragments as a function of BTA concentration is shown in Figure 18. The fragment Cu_2_Cl^+^ apparently shows a similar distribution as CuCl_2^−^_ and corresponds to the chloride ions on the copper surface.

The distribution of Cu_4_(BTA)_3^+^_ is very similar to that of the negative fragment C_12_H_8_N_6_Cu^−^. It means that these two fragments correspond to a similar parental molecular arrangement. It supports the proposed Cu_4_(BTA)_3^+^_ molecular structure (Figure 2), in which the central copper core is covered by the BTA-Cu-BTA and one free BTA molecule. On the other hand, binding of BTA-Cu-BTA and two BTA molecules to the copper core in the form Cu_5_(BTA)_4^+^_ is possible only at 30 and 50 ppm of BTA if all chloride ions are removed from the copper surface. No chloride complexes such as Cu_4_(BTA)_3_CuCl^+^ with mass 705.80 identified in the dip-coating experiments (Section 2.1) for samples 1 and 11 were observed in the positive ion mode mass spectra. It means that such complexes can be formed in bulk solutions under chemical conditions in less acidic solutions, as was conducted in the dip-coating experiment.

### 2.8. Incorporation of BTA into Copper during Copper Electrodeposition from Electrolyte Containing BTA with Chloride Ions—TOF-SIMS Measurements

**Figure 19:**
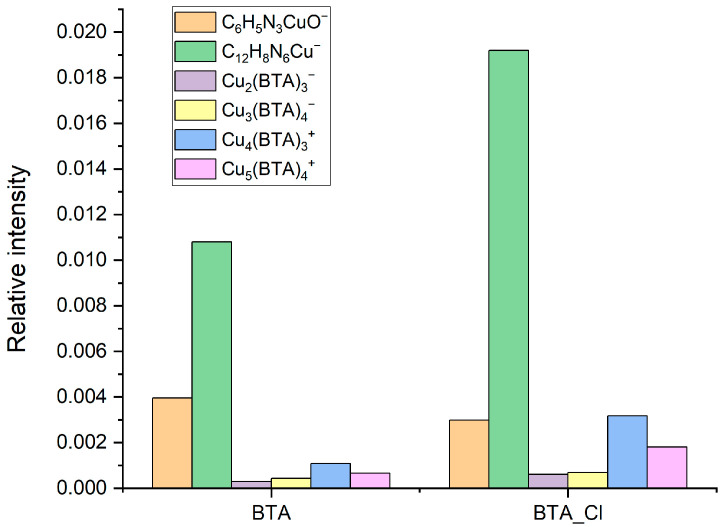
Intensity distribution of C_6_H_4_N_3_CuOH^−^, C_12_H_8_N_6_Cu^−^, Cu_2_(BTA)_3^−^_, Cu_3_(BTA)_4^−^_, Cu_4_(BTA)_3^+^_, and Cu_5_(BTA)_4^+^_ on the copper surface after etching in nitric acid. For BTA 50 ppm and chloride ions (right plot). For comparison, the same fragments for BTA at 50 ppm without chloride ions are shown on the left side.

The incorporation of the selected monomeric, dimeric, trimeric, tetrameric, and pentameric forms into the copper deposit is shown in Figure 19. It is clearly seen that for 50 ppm of BTA with chloride ions (right side of plot), the contribution of the incorporated dimeric copper complex form C_12_H_8_N_6_Cu^−^ in relation to the monomeric form C_6_H_4_N_3_CuOH^−^ is higher than it was in BTA without Cl (left plot). Moreover, the amount of incorporated polymeric Cu_4_(BTA)_3^+^_ and Cu_5_(BTA)_4^+^_ forms is also greater than it was without chloride ions. It means that chloride ions stabilize the incorporation of organometallic complexes of BTA into deposits. It can be determined by the lower mobility of the Cu-BTA complexes in the presence of Cu-BTA-Cl. Moreover, the surface roughness in the presence of chloride ions may significantly change, which may increase the adsorption energy of copper metallic complexes in situations where the number of coordinated copper atoms decreases [28,87]. Surface roughness with and without chloride ions was examined by atomic force microscopy (Section 2.11).

Overall, in the presence of chloride ions, BTA molecules are incorporated into copper deposits similarly to what was shown in Figure 12. While partial desorption of incorporated BTA into copper deposits was postulated previously [21]. Incorporation of BTA molecules and chloride ions by examination of Cl and S by means of TOF-SIMS in dynamic mode was carried out previously [77].

### 2.9. Distribution of BTA and Micromorphology of Copper Surface Obtained from Electroplating Bath with Chloride Ions

**Figure 20:**
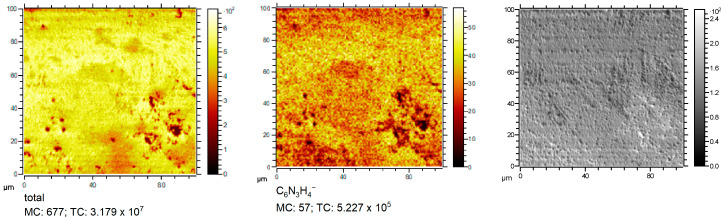
Distribution of total ion image (left) and C_6_H_4_N_3^−^_ (middle) for BTA 50 ppm/Cl 30 ppm at wire position 1900 µm. Scanning area: 100 × 100 µm. The right column image shows a backscattered secondary electron image with a resolution of 0.2 µm. The dark areas in the total ion image correspond to the low amount of BTA as depicted by the C_6_H_4_N_3^−^_ ion distribution. The dark areas in the total ion image and C_6_H_4_N_3^−^_ correspond to the crystals visible in the SE image.

Figure 20 depicts the distribution of the total ion image, C_6_H_4_N_3^−^_, and the backscattered secondary electron image. The darker areas in the total ion image correspond to the low amount of BTA (C_6_H_4_N_3^−^_).

The secondary electron image (right image) shows that crystals appear in areas corresponding to very low BTA surface coverage. Such observations were also noticed for BTA without chloride. It proves the inhibition properties of BTA in suppressing copper crystal growth at the microscale. Investigation of BTA influence on surface morphology at the nanoscale for all BTA concentrations in the presence of chloride ions was examined by AFM (results and discussion in Section 2.11).

### 2.10. Chemistry of Copper Surface Deposited from Electrolytes Containing BTA with Chloride Ions—SERS Studies

**Figure 21:**
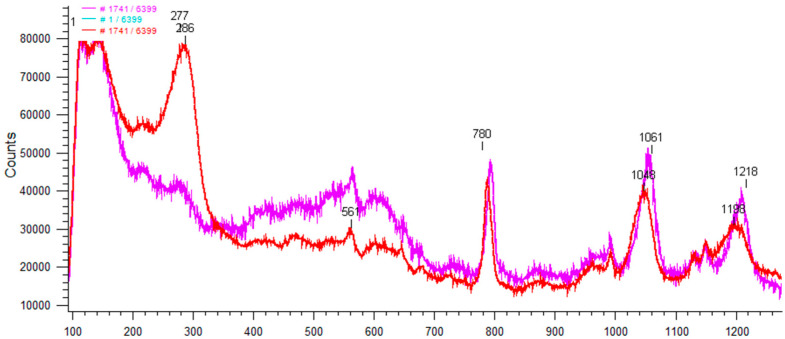
SERS spectra for BTA without chloride ions (pink) and with chloride ions (red color).

After the addition of chloride ions, a few significant differences in the SERS spectra in comparison to the samples without chloride ions (Section 2.7) can be observed (Figure 21).

**Figure 22:**
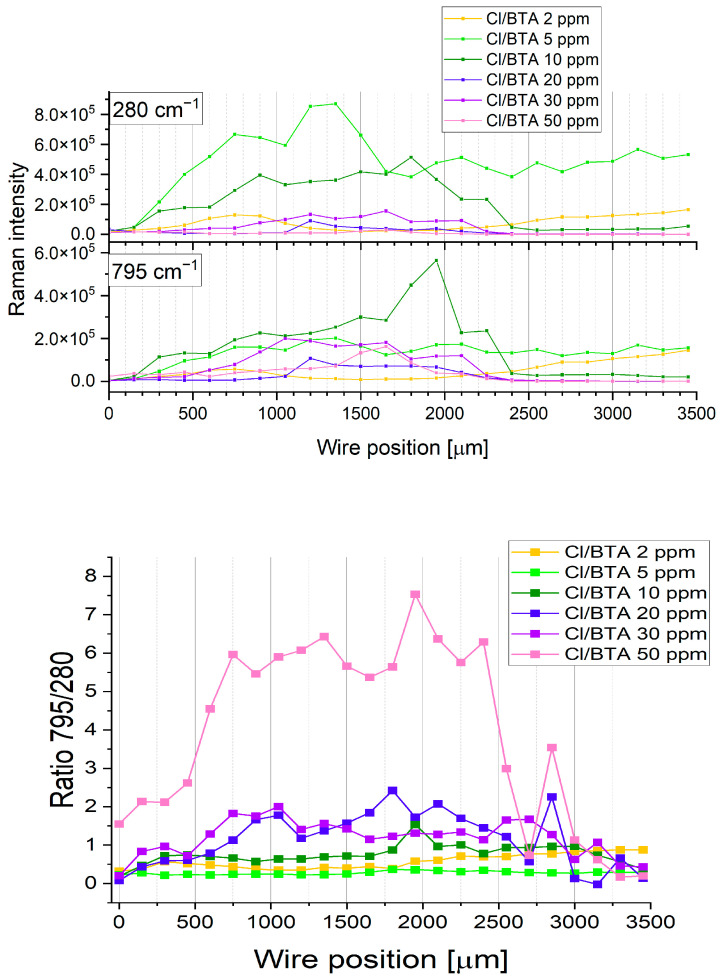
Distribution of Raman intensity for bands 280 cm^−1^, 795 cm^−1^, and the ratio bands 795/280 along wire position for 2, 5, 10, 20, 30, and 50 ppm of BTA in the presence of Cl (30 ppm) in the solution that shows chloride replacement by BTA molecules (see for comparison Figure 15 that presents a similar effect examined by TOF-SIMS).

A strong, wide band at 280 cm^−1^ corresponds to the Cu-Cl bond [92]. Moreover, the bands 795 cm^−1^ and 1053 cm^−1^ are slightly redshifted, c.a. 3 cm^−1^. The intensity of the band 1053 cm^−1^ is significantly reduced, and the peak is broadened. Furthermore, the band 1209 cm^−1^ characteristic for the molecular form of BTAH corresponds to the adsorbed BTA molecules on metallic copper and is significantly reduced and redshifted to 1193 cm^−1^. The distribution of bands 300 cm^−1^ and 800 cm^−1^ at 0, 2, 10, 20, 30, and 50 ppm of BTA with the presence of 30 ppm chloride ions along the wire position is shown in Figure 22.

Raman intensity of band 280 cm^−1^ is not inversely proportional to the BTA concentration as it was observed for TOF-SIMS (see Figure 15 and Figure 16, CuCl_2^−^_, and Figure 18 Cu_2_Cl^+^). We should expect the greatest Raman intensity for the Cu-Cl bond at 0 ppm of BTA, whereas the lowest is at 50 ppm of BTA. As it is seen in Figure 22, the greatest SERS intensity is observed at 5 ppm of BTA, while the lowest is at 50 ppm. It arises from the fact that the SERS signal is very sensitive to the surface roughness [84,85] and thickness of the layer, as shown in Section 2.5.

Similarly, for band 795 cm^−1^ (benzene ring breathing in BTA), one can observe the greatest Raman intensity increases for BTA concentrations from 2 to 10 ppm, while for greater BTA amounts, the Raman signal is significantly reduced in rather random order. It is contrary to the TOF-SIMS, with the intensity of fragments characteristic for BTA proportionally increasing with the BTA amounts in the solution (Figure 15, Figure 16 and Figure 18). Figure 22 (bottom plot) shows the ratio of bands 795/280 for 0, 2, 10, 20, 30, and 50 ppm of BTA that corresponds to the ratio BTA/Cl on the copper surface and illustrates the replacement of chloride ions by BTA molecules.

The ratio 795/280 slowly increases for BTA concentrations from 2 to 10 ppm. Subsequently, significant increases occurred for 20 and 30 ppm of BTA. For the highest BTA amount (50 ppm), the ratio is very high (about 6) in comparison to the rest of the samples. It may suggest that all chloride ions are replaced by BTA molecules.

**Figure 23:**
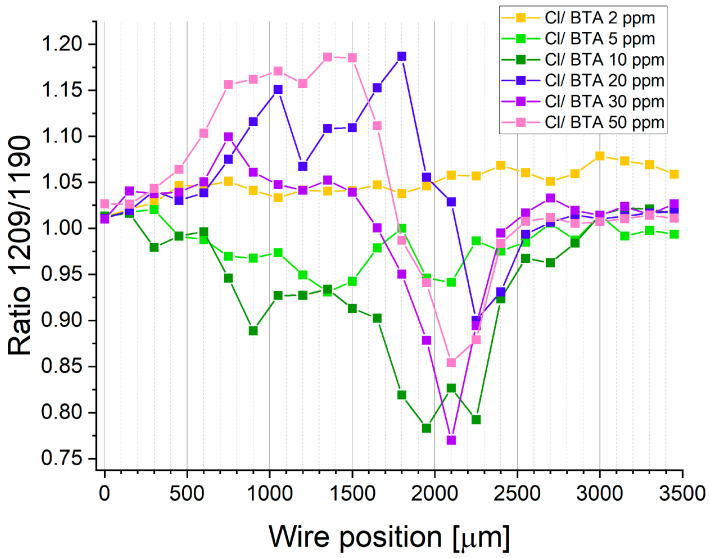
Ratio of intensity bands 1208/1190 for different BTA concentrations.

To estimate possible forms of BTA (molecular or complex) on the copper, we calculated the 1208/1190 ratio similarly to how it was conducted for BTA without chloride ions (Figure 23).

At 2 ppm of BTA, the 1208/1190 ratio is constant for the whole potential range. It means that the shape of band 1028 cm^−1^ is not changing and that BTA exists only in molecular form. For 5 and 10 ppm of BTA, the ratio 1208/1190 diminishes for forward scan (wire position 0–1500 µm). For reverse scan, for 5 ppm, the ratio 1208/1190 is varied, while for 10 ppm of BTA, it is further reduced up to wire position 2300. When the SERS signal is significantly reduced (wire position 2400–3500 µm), the ratio 1208/1190 for 10 ppm of BTA increases. It may suggest that for high overpotentials of reverse scanning when strong desorption of BTA and Cl ions occurs, the molecular form of BTA dominates over the complex form of BTA due to the dissolution of the Cu(I)BTA complex by hydrogen ions.

A similar observation for high overpotential was previously noticed for BTA on copper surfaces in sulfuric acid [63]. For 30 ppm of BTA, the ratio 1208/1186 is rather constant for the forward scan, while for 20 and 50 ppm of BTA, it is significantly higher. After switching the sweep direction from anodic to cathodic, the ratio 1208/1190 significantly diminishes for 20, 30, and 50 ppm of BTA up to wire position 2100–2300 µm. It corresponds to the region when the SERS signal is strongly reduced (Figure 22). Overall, only for 2 and 5 ppm of BTA, we could not observe such a significant decrease in the ratio 1208/1190. However, for these samples, the Raman signal does not decrease at the wire position from 2400 to 3500 µm. Moreover, we can conclude that the ratio of the molecular form of BTA to the complex form, expressed as a ratio of 1208/1190, dominates for BTA at 20 and 50 ppm, while the complex form may coexist with the molecular form at 5 and 10 ppm for forward scanning.

The Cu(I)BTACl complex formation can be driven by the coexistence of Cl and BTA species as well as the optimal molar ratio of BTA/Cl on the copper surface. The existence of the complex form Cu(I)BTACl is noticed at low ratios of BTA/Cl observed for 5 and 10 ppm of BTA (ratio 795/280, Figure 22).

On the other hand, at a lower amount of BTA (2 ppm), the Cu-Cl complex formation dominates, and the formation of the Cu-BTA-Cl complex does not occur. For BTA concentrations greater than 10 ppm, the ratio BTA/Cl significantly increases (Figure 22), which shows a reduced available number of chloride ions near the BTA molecules, remarkably decreasing the probability of Cu-BTA-Cl complex formation.

However, the SERS data does not support the existence of complex forms for concentrations greater than 20 ppm of BTA. On the other hand, the existence of C_12_H_8_N_6_Cu_2_Cl^−^ was proven by TOF-SIMS, which provides direct information about molecular species in contrast to the SERS, where assignments of bands are very often not certain and the abundance of different bands is very sensitive to the surface roughness and thickness of the deposited layer, as shown in Section 2.5 and in this section.

### 2.11. Surface Morphology at the Nanoscale—Atomic Force Microscopy Measurements

**Figure 24:**
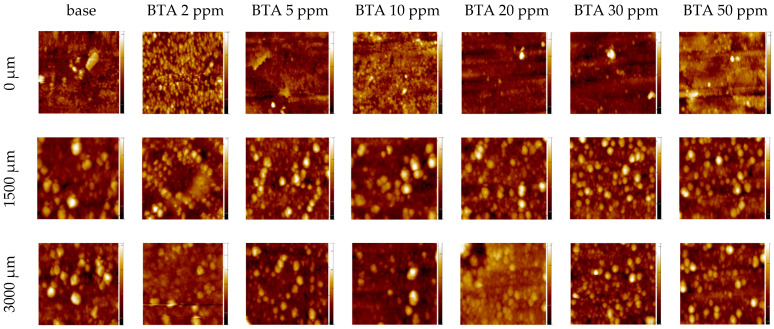
AFM micrographs of copper surfaces electrodeposited from solution contained: 0, 2, 5, 10, 20, 30, and 50 ppm of BTA at wire positions of 0, 1500, and 3000 µm. Moreover, 0 µm corresponds to the copper surface at the starting point of the cyclic voltammetry experiment (potential −0.6 V forward scan), position 1500 µm corresponds to the OCP, and 3000 µm corresponds to the −0.6 V overpotential—reverse scan. Scanning area: 20 × 20 µm.

The micrographs of the copper surface obtained with the presence of BTA in the electroplating solution without chloride ions are shown in Figure 24.

Position 0 µm corresponds to the starting point of the CV experiment at an overpotential −0.6 V. The copper layer was obtained at this point by electrodeposition at a constant current density −15 mA/cm^2^ for 120 s. The surface roughness parameter (Sq) is included in Table 4.

After the addition of 2 ppm BTA, roughness is significantly reduced from 79 nm to 52 nm. The roughness obtained for 2 and 5 ppm of BTA was remarkably lower than for 20, 30, and 50 ppm of BTA. It seems that the lower levels of BTA molecules on the copper and into the copper deposit observed at 2 and 5 ppm demonstrate the strongest inhibiting properties for copper crystal growth. At higher amounts of BTA (10, 20, 30, and 50 ppm), the excess of accumulated BTA dimeric and polymeric forms perturbs the diffusion control of copper ions onto the copper surface. Moreover, polymeric complexes of Cu_4_(BTA)_3^+^_ and Cu_5_(BTA)_4^+^_ identified in the TOF-SIMS spectra consist of core copper clusters that can induce agglomeration of copper adatoms. Similar effects can occur for dimeric and linear forms of Cu-BTA complexes. During the CV experiment (positions 1500 and 3000 µm), roughness rapidly grows, while under the OCP condition (wire position 1.5 mm), the lowest roughness is observed for 30 and 50 ppm of BTA. Rapid growth of surface roughness during the CV experiment is determined mainly by the fact that at high overpotential conditions during forward scanning, BTA is not present or occurs in very low amounts on the surface (notice TOF-SIMS data, Figure 5). Under these circumstances, copper grain growth is not inhibited. It means that BTA influence on the morphology strongly depends on applied overpotential. During reverse scan at −0.6 V (wire position 3000 µm), for low BTA concentrations (2 and 5 ppm) and the greatest (30 and 50 ppm), the surface roughness is maintained, while for 10 and 20 ppm, it is varied. Similarly to the results we observed at the 0 µm wire position, copper electrodeposited from solutions consisting of BTA demonstrated significant brightening abilities in comparison to the base electrolyte [21,22,23,66].

**Figure 25:**
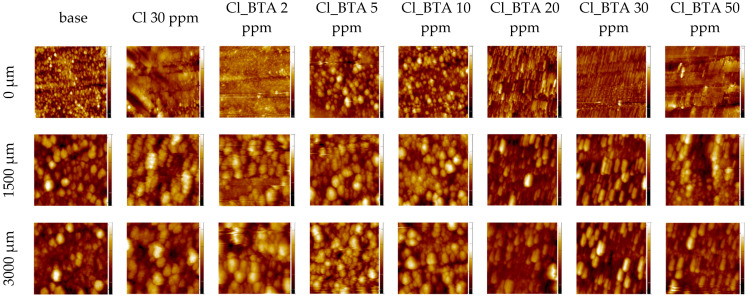
AFM micrographs for copper surfaces electrodeposited from solution contained: 0, 2, 5, 10, 20, 30, and 50 ppm of BTA and 30 ppm of chloride ions at wire positions of 0, 1500, and 3000 µm. Moreover, 0 µm corresponds to the copper surface at the starting point of the cyclic voltammetry experiment (potential −0.6 V, forward scan); position 1500 µm corresponds to the OCP; and 3000 µm corresponds to the −0.6 V overpotential, reverse scan. Scanning area: 20 × 20 µm.

After the addition of chloride ions, surface morphology significantly changes (Figure 25). At wire position 0 µm, which corresponds to the copper electrodeposited at −15 mA/cm^2^, the surface contains numerous crystallites from the base electrolyte and roughness at a level of 35 nm (Table 5). After injection of chloride ions, the surface considerably refines to develop a noninotropic structure with large flat terraces (Sq = 41 nm). After the addition of BTA (2 ppm), a significant number of small grains appear on the surface while roughness increases. The next portion of BTA (5 ppm) determines the increasing size of copper protrusions. In consequence, the surface roughness increases (Sq = 100 nm). At 10 ppm of BTA, due to the significant replacement of chloride ions (see TOF-SIMS results) by BTA molecules, the process of grain growth is inhibited and roughness is reduced (Sq = 67 nm). For 20 ppm, the characteristic lamellar forms appear with a lower diameter than the grains noticed at 10 ppm BTA. In effect, a lower roughness (54 nm) was observed. The process is constantly intensified at 30 ppm of BTA, where lamellar forms demonstrate smaller diameters and lower surface roughness (38 nm). At the greatest BTA amount (50 ppm), lamellar forms disappear (Sq = 41 nm). During the CV experiment under the OCP regime (wire position 1500 µm), surface roughness dramatically increased for all samples (Table 4). All characteristic features distinguished on the copper surface at position 0 µm are also observable and growing. A similar effect is observed at the greatest overpotential for reverse scan (−0.6 V, wire position 3000 µm). In the latter case, it is obvious that the time of electroplating during the CV experiment (30 s from OCP to −0.6 V) is not sufficient to refine the morphological structure of the copper deposit determined at OCP. On the other hand, during the same time electrodeposition, the potential sweep from −0.6 V to OCP (wire position from 0 to 1500 µm) changes the morphology and structure. It seems that the formation of the complex Cu-BTA-Cl identified in the TOF-SIMS spectra is responsible for the significant structure changes. Characteristic lamellar structures at 20, 30, and 50 ppm were formed as a significant amount of the chloride complex C_12_H_8_N_6_Cu_2_Cl^−^ was identified (see Section 2.7).

Evaluation of surface roughness for 10 µm thick of copper deposited from solutions BTA (12 ppm) and BTA (12 ppm)/Cl^−^ (10 ppm) at −40 mA/cm^2^ at a rotating speed of 5000 rpm showed [21] that after addition of Cl^−^ ions, roughness rapidly grows. In our experimental conditions at a comparable molar ratio of BTA (30 ppm)/Cl^−^(30 ppm), we observed that roughness is lower than it was for BTA (30 ppm) only. However, we electrodeposited copper at a lower current density (i.e., −15 mA/cm^2^), the electroplating bath was not stirred, and the thickness was around 2 µm.

## 3. Materials and Methods

The dip-coating experiment (Section 2.1) was carried out in a test tube containing 10 mL of electrolyte. During a dip-coating experiment, 20 samples were prepared (Table 1). The nitinol wire (assigned as Sample 1, NiTi) was immersed for 60 s in a solution containing 0.1 M BTA/6.25mM CuCl_2_ and then withdrawn at a constant speed of 1 mm/s and dried in the air at room temperature. The next sample (2, NiTi/rinsing) was prepared in the same way, but after deposition, it was rinsed in a 5% ethanol solution. The procedure for rinsing was described in the experimental section. Similarly, the subsequent nitinol samples were prepared in the same way, while the composition of additives varied. The same procedure of deposition was applied to the nitinol wires with a pre-plated copper layer.

Samples were assigned as: NiTi; NiTi/rinsing; Cu; Cu/rinsing; Cu; and Cu/rinsing, depending on the method of preparation.

The base electrolyte contained sulfuric acid (99.9% pure, POCH S.A., Gliwice, Poland) and copper sulfate (99% pure, Supelco, Bellefonte, PA, USA). The following additives were added: Cl^–^ (in the form of HCl, POCH S.A., Gliwice, Poland) and benzotriazole (99% pure, Sigma-Aldrich, Saint Louis, MO, USA). For the preparation of all solutions, deionized water (18 MΩ, Hydrolab, Straszyn, Poland) was applied. For cyclic voltammetry experiments (CV), a 120 mm piece of superelastic nitinol wire (0.665 mm diameter, Ni—55%, Ti—45%, supplied by Euroflex GmbH, Pforzheim, Germany) was immersed in a copper sulfate solution for a length of 50 mm (1 cm^2^ surface area).

Cyclic voltammetry (CV) experiments were accompanied by TOF-SIMS experiments, and a copper ring with phosphor as an additive (45 mm outside and 30 mm internal diameter, 1 mm thickness, supplied by Lesker, Jefferson Hills, PA, USA) was employed as counter electrodes and mounted on the bottom of the test tube. The tube contained 250 mL of the electroplating bath. The purity and manufacturer of components of the electroplating bath were mentioned above, while the concentration of the base solution (without additives) was as follows: CuSO_4_* × 5H_2_O—0.225 M, sulfuric acid—0.56 M. Accordingly, benzotriazole at concentrations of 0, 2, 5, 10, 20, 30, and 50 ppm without and with Cl^−^ ions, at a concentration of 30 ppm, was added.

A counter-electrode was connected to a galvanostat using a copper wire (diameter 1 mm) covered by a thick isolated lacquer layer, resisting the electroplating bath. As a reference electrode, an Ag/Ag_2_SO_4_/(sat.)K_2_SO_4_ electrode (Eurosensor, Gliwice, Poland) was applied. Reference potential (vs. SCE) was equal to +0.163 V (according to the manufacturer’s statement). A galvanostat AUTOLAB 302N (Eco Chemie, Utrecht, The Netherlands) and software NOVA 1.11 (Methrohm, Utrecht, The Netherlands) were employed for cyclic voltammetry (CV) experiments. The ohmic overpotential estimated at 10 Ω during CV scans was not corrected. Before the addition of sulfuric acid and additives, the copper sulfate solution was thoroughly deoxygenated using an ultrasonic washer under reduced pressure. The conductivity of the base solution was equal to 208 mScm^−1^ at pH 1.05, respectively. The solution temperature was equal to 23 °C. The electroplating bath was not stirred. Before each experiment, the counter-electrode was immersed in fresh 30% HNO_3_ for 30 s, then thoroughly rinsed in deionized water and dried in the air. Subsequently, nitinol wire, which served as a working electrode, was mounted vertically in the electroplating bath, and copper was electrodeposited at a constant current density −15 mA/cm^2^ for 120 s. After copper preplating, nitinol wire with a copper layer was withdrawn at a constant speed equal to 34 µm/s from the copper electroplating bath under electrodeposition conditions by means of an upright-mounted motorized ES111 OptiScan stage (Prior Scientific Instruments Ltd., Cambridge, UK) Simultaneously, the CV experiment was performed, and the applied potential was swept at constant velocity, V = 20 mV/s, in the range of overpotential between −0.6 V and OCP (forward scan to anodic direction) and from OCP to −0.6 V (reverse scan to cathodic direction), where OCP is an open circuit potential. OCP was determined for 120 s or less before the CV experiment on the copper preplated layer until a steady state was reached. In this manner, during a CV experiment, one cyclic loop corresponds to 60 s (30 s for the forward scan in the anodic direction and 30 s for the reverse scan in the cathodic direction) and 2 mm of the nitinol wire (working electrode) length during a withdrawal. The forward scan from −0.6 V to OCP corresponds to the wire position from 0 to 1000 µm, while the reverse scan corresponds to the position from 1000 to 2000 µm, respectively. An additional scheme of the CV experiment is shown in Figure S3 (Supplement Materials). To avoid copper sulfate salting out, an optimized method of rinsing was applied. More details on the rinsing method can be found elsewhere [19]. Briefly, a glass capillary with an internal diameter of 2.4 mm was immersed in a rinsing solution (5% aquatic ethanol) and mounted horizontally. Deionized water for preparing the rinsing solution (5% aquatic ethanol) was boiled to remove carbon dioxide, which may increase the solubility of the examined solid films adsorbed on the copper surface during electrodeposition. The total volume of rinsing solution inside the capillary was 8 µL (height of rinsing liquid: ~1 mm) and was collected at the bottom end of the capillary. The rinsing properties of 5% ethanol in deionized water and other concentrations of ethanolic solutions were determined in previous work [43].

After preparation, samples were transferred to the TOF-SIMS vacuum chamber and routinely measured not later than 2–5 h after preparation. TOF-SIMS spectra were acquired by means of the TOF-SIMS.5 instrument (ION-TOF GmbH, Münster, Germany). The primary ion source of Bi^+^ was used at 30 keV (cyclic time 100 µs, primary beam current 1.2 pA). The scanning area of the secondary ions was 50 × 50 µm with 128 × 128 pixels and 1 shot/pixel. All the measurements were performed in a static mode (dose no larger than 1 × 10^12^ ions/cm^2^) in a negative mode. For most of the samples, 30 or more points separated by 0.1 mm were measured. The post-processing data analysis was conducted using the SurfaceLab 6.7 software (ION-TOF) and Origin 2019 (OriginLab, Northampton, MA, USA). The negative spectra were recorded and calibrated using the positions of CH^−^, CH_2^−^_, and CH_3^−^_. Intensities were normalized to the total intensity.

After TOF-SIMS measurements, samples were taken for SERS measurements. SERS spectra were acquired by means of Raman via microscopy (Renishaw, Sheffield, UK). Measurements were carried out using a 785 nm laser source with a power of 50 mW on the sample (100% power), an exposure time of 50 ms, and one acquisition in StreamLineHR image acquisition mode with a step of 5um with an Andor detector. Objective 50× was carried out. The post-processing data analysis was conducted using the Wire 4.3 software (Renishaw) and Origin 2019. Subsequently, samples were transferred for atomic force microscopy. Measurements were carried out by atomic force microscopy with an Agilent 5600LS (Santa Clara, CA, USA) in tapping mode with a silicon tip (radius 7 nm). The data were evaluated using SPIP 5.1.4 (Image Metrology, Kongens Lyngby, Denmark).

## 4. Conclusions

In this paper, we examined for the first time the influence of benzotriazole on the copper electrodeposition with and without the presence of chloride ions by combining studies of cyclic voltammetry, TOF-SIMS, SERS, and AFM.

The obtained results show that benzotriazole, after dissociative adsorption to the copper surface, reduces current density in a logarithmic way as a function of BTA concentration for the studied concentration range of 2 to 50 ppm.

It was identified that the characteristic Cu-BTA fragments exist in negative and positive modes. The most prominent forms of Cu-BTA adducts identified in the negative mode correspond to the monomeric hydroxylated form of Cu-BTA in the form of C_6_H_4_N_3_CuOH^−^, the dimeric organometallic complex consisting of copper adatom, C_12_H_8_N_6_Cu^−^, and the trimeric form, C_18_H_16_N_9_Cu_2^−^_. All forms identified in the negative modes demonstrate linear structure. In the positive mode, the trimeric and tetrameric forms Cu_4_(BTA)_3^+^_ and Cu_5_(BTA)_4^+^_ exist in cyclic forms that surround the central copper core.

In the presence of chloride ions at a concentration of 30 ppm, the inhibiting properties of BTA diminish due to the existence of Cl ions on the copper surface. Chloride ions are gradually desorbed. At BTA concentrations of 30 and 50 ppm, no chloride ions were directly adsorbed on the copper surface, while Cu-BTA-Cl complex formation was proven by TOF-SIMS and indirectly by SERS studies. The Cu-BTA-Cl complex was identified in the form of C_12_H_8_N_6_Cu_2_Cl^−^ and C_6_H_4_N_3_Cu(I)Cl. We proposed a scheme of formation for the Cu-BTA-Cl complexes.

Incorporation of BTA molecules into copper deposits, mainly in dimeric and polymeric forms, was proven for BTA with and without chloride ions in the electroplating bath.

It was also shown that the distribution of BTA spices on the copper electrode correlates with the microroughness of copper surfaces identified by backscattered electrons.

Roughness at the nanoscale was examined by atomic force microscopy and showed the existence of a specific lamellar structure on the copper surface in the presence of chloride ions. However, the structure strongly depends on the applied potential and composition of the electroplating solution.

## Figures and Tables

**Table 1:** The assignment of samples that were obtained by the dip-coating experiment. The scheme of assignments: NiTi—nitinol wire immersed for 60 s; NiTi/rinsing—nitinol wire immersed for 60 s and then rinsed; Cu—nitinol wire pre-plated by copper and immersed for 60 s; Cu/rinsing—nitinol wire pre-plated by copper immersed for 60 s and then rinsed.

	NiTi	NiTi/Rinsing	Cu	Cu/Rinsing
0.1M BTA/6.25 mM CuCl_2_	1	2	11	12
0.1M BTA/6.25mM CuCl_2_/0.1M H_2_SO_4_	3	4	13	14
0.1M BTA/0.1M Cu(NO_3_)_2_	5	6	15	16
1.25 mM BTA/0.78mM CuSO_4_	7	8	17	18
0.39 mM BTA/0.39mM CuCl	9	10	19	20

**Table 2:** The *m*/*z* assignment of negative and positive fragments identified in the TOF-SIMS mass spectra.

No.	Center Mass (u)	Assignment
1	62.99	Cu^−^
2	76.07	C_6_H_4^−^_
3	90.08	C_6_H_4_N^−^
4	94.98	CuO_2^−^_
5	95.99	SO_4^−^_
6	97.00	HSO_4^−^_
7	97.95	CuCl^−^
8	118.11	C_6_N_3_H_4^−^_
9	123.96	CuCNCl^−^
10	132.93	CuCl_2^−^_
11	181.09	C_6_H_4_N_3_Cu^−^
12	198.11	C_6_N_3_H_5_CuO^−^
13	207.13	C_6_H_4_N_3_CuCN^−^
14	216.10	C_6_H_4_N_3_CuCl^−^
15	251.12	C_6_H_4_N_3_CuCl_2^−^_
16	299.25	C_12_N_6_H_8_Cu^−^
17	314.02	C_6_H_4_N_3_Cu_2_Cl_2^−^_
18	388.26	C_6_H_4_N_3_C_6_H_4_N_3_Cu_2_CN^−^
19	397.24	C_6_H_4_N_3_C_6_H_4_N_3_Cu_2_Cl^−^
20	480.40	C_6_H_4_N_3_C_6_H_4_N_3_C_6_H_4_N_3_Cu_2^−^_
21	62.93	Cu^+^
22	125.86	Cu_2^+^_
23	153.86	Cu_2_N_2^+^_
24	160.83	Cu_2_Cl^+^
25	243.92	Cu_2_C_6_H_4_N_3^+^_
26	605.89	Cu_4_(C_6_H_4_N_3_)_3^+^_
27	705.80	Cu_4_(C_6_H_4_N_3_)_3_H_2_CuCl^+^
28	786.87	Cu_5_(C_6_H_4_N_3_)_4^+^_

**Table 3:** Raman shift and assignment for the most prominent bands identified in the Raman and SERS spectra shown in Figure 11.

Solid BTA	Cu(Poly) [63]	Roughened Cu(Poly) [63]	Chan and Weaver [64]	Band Assignments
429	435	436	440	Skeletal torsion
537	549	553	555	Triazole ring bend
628	630	635	653	Triazole ring torsion
779	785	788	790	Benzene ring breathing
	982	974		SO_4_ symmetric stretch
1006	1019		1010	Benzene skeletal and (CH) bend
1022	1025	1024	1020	Benzene skeletal and (CH) bend
1072				(NH) bend
1127	1128	1127		(CH) bend
1147	1134	1145	1140	(NH) bend
1171	1164	1159	1160	Triazole asymmetric stretch and (NH) bend
1206	1193	1196	1190	Triazole-ring breathing mode (C–C–C in-plane bending)
1280	1286	1284	1286	Skeletal stretch (NH) bend and (CH) bend
1374	1377	1380	1370	Fermi resonance with 1385
1387	1388	1391	1385	Combination breathing
	1444		1440	Skeletal stretch

**Table 4:** Surface roughness (Sq) determined for copper layer obtained from solution containing 0, 2, 5, 10, 20, 30, and 50 ppm of BTA at wire positions: 0, 1500, and 3000 µm.

Wire Position	Base	BTA 2 ppm	BTA 5 ppm	BTA 10 ppm	BTA 20 ppm	BTA 30 ppm	BTA 50 ppm
0 µm	79.124	52.454	33.264	34.067	42.567	49.747	45.719
1500 µm	140.19	96.149	87.30	130.58	95.958	80.233	83.440
3000 µm	100.76	96.564	91.148	87.468	118.87	81.827	82.237

**Table 5:** Surface roughness (Sq) determined for copper layer obtained from solution containing: 0, 2, 5, 10, 20, 30, and 50 ppm of BTA with the presence of 30 ppm of chloride ions at wire positions: 0, 1500, and 3000 µm.

Wire Position	Base	Cl 30 ppm	Cl_BTA 2 ppm	Cl_BTA 5 ppm	Cl_BTA 10 ppm	Cl _BTA 20 ppm	Cl_BTA 30 ppm	Cl _BTA 50 ppm
0 µm	35.409	41.525	60.579	100.76	67.580	53.984	38.826	41.014
1500 µm	142.89	245.31	217.50	191.20	162.27	126.51	145.92	167.15
3000 µm	134.38	252.31	252.33	183.81	202.65	209.98	153.11	127.71

## Data Availability

The TOF-SIMS, AFM, CV, and Raman data can be obtained upon reasonable request.

## Supplementary Materials

The following supporting information can be downloaded at: https://www.mdpi.com/article/10.3390/molecules28155912/s1.

## Sample Availability

Samples of the compounds are not available from the authors.
