# Supplementary material for: Studies of Benzotriazole on and into the Copper Electrodeposited Layer by Cyclic Voltammetry, Time-of-Flight Secondary-Ion Mass Spectrometry, Atomic Force Microscopy, and Surface Enhanced Raman Spectroscopy
Source: Molecules. 2023 Aug 6;28(15):5912. doi: 10.3390/molecules28155912 (PMC10420986; doi:10.3390/molecules28155912)
Supplement: Supplementary file 1 [file molecules-28-05912-s001.zip › molecules-2526194-supplementary.pdf]

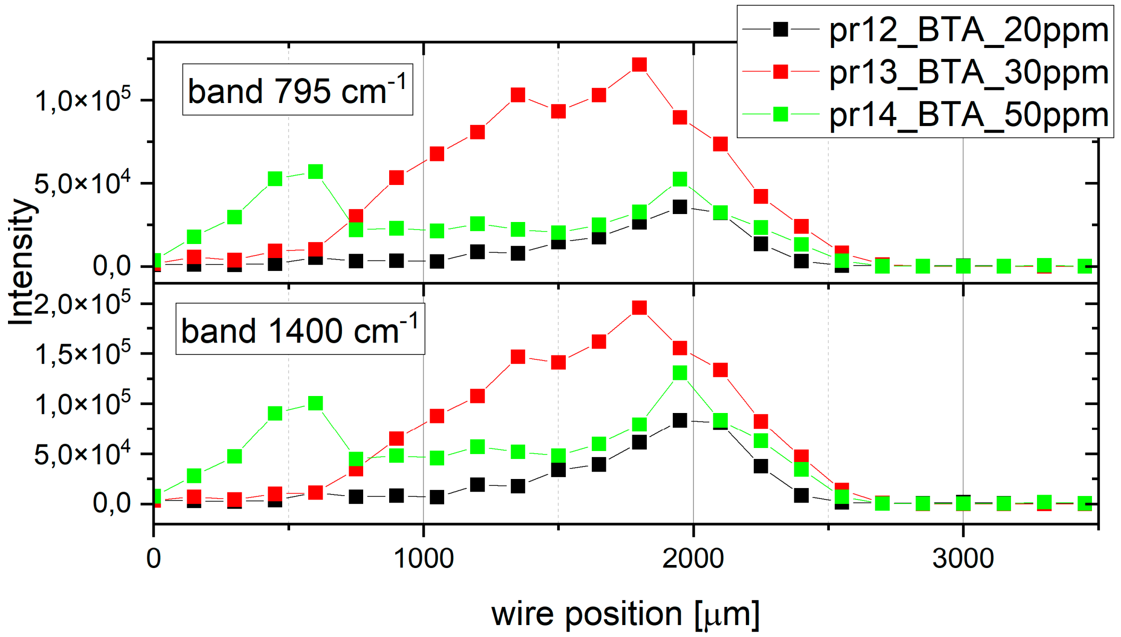

Figure S1. Raman intensity distribution for band 795 and 1400 for 20, 30 and 50 ppm of BTA in a function of wire position. Significant reduction of intensity for 50 ppm is seen for the wire position form 750 to 2000 um.

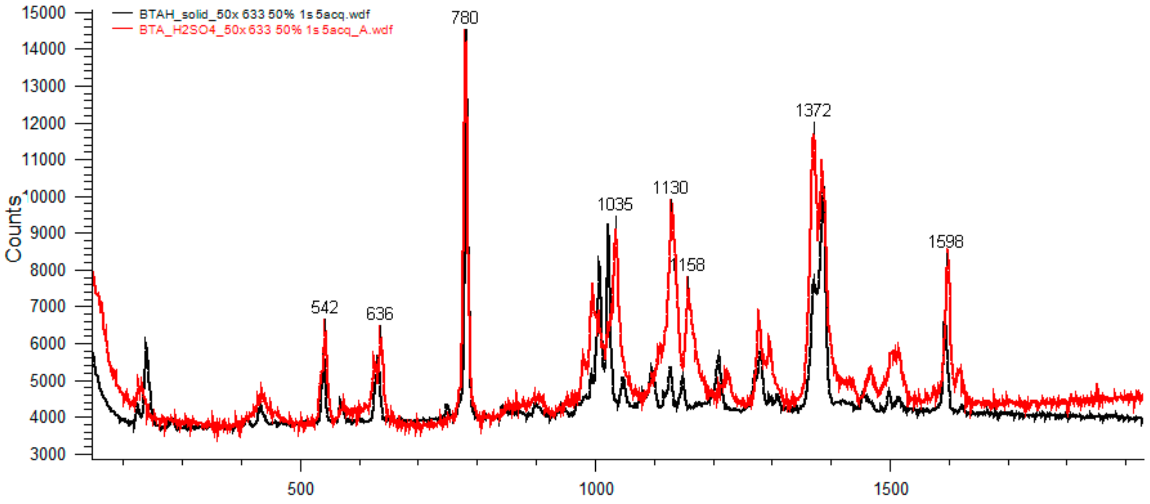

Figure S2 Normal Raman spectra for solid BTAH (black) and solid blue crystal Cu(II)$BTA_4SO_4H_2O$ prepared as it was described in [1]. X-ray structural data can be find elsewhere [2]

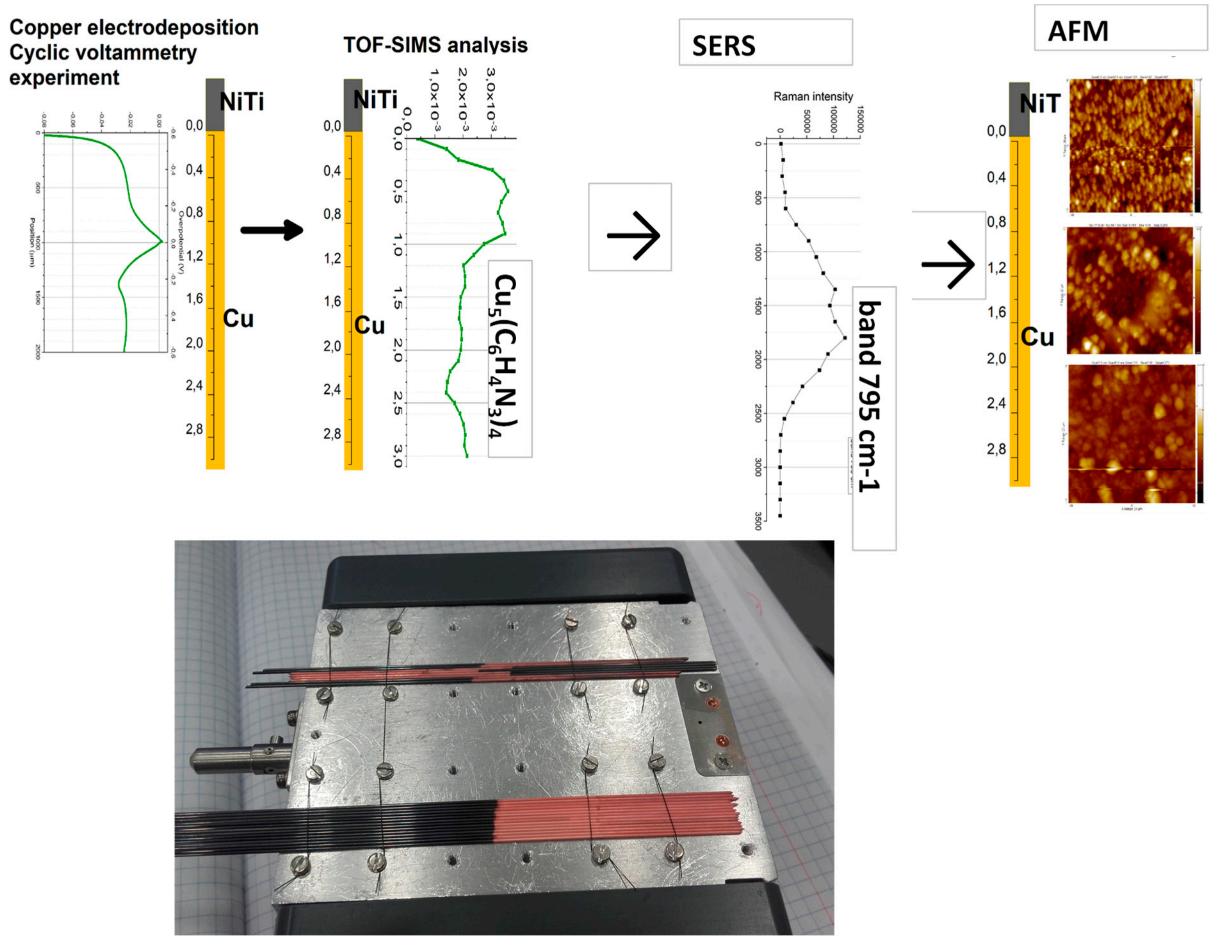

Figure S3. The scheme of experiments: cyclic voltammetry was carried out by withdrawal of vertically mounted nitinol wire immersed into electroplating bath. After deposition and rinsing electroplated nitinol wires were mounted onto special stage holder (bottom image) and transferred to TOF-SIMS instrument. Consecutively to SERS and AFM analysis. SERS and AFM analysis were carried out without removing the electroplated wires from stage holder.

1. Kawecka Z.; Pajdowski, L. Transition Metal Complexes with Nitrogen - Containing Ligands P.1. Copper /I- Benzotriazole Complexe. *Roczniki chemiczne* **1976**, *50*, 1251–1256.
2. Hursthouse, M.B.; Short, R.L.; Robinson, S.D. X-Ray Crystal and Molecular Structure of Sulphato Aquotris(Benzotriazole) Copper(II)Benzotriazole. *Polyhedron* **1986**, *5*, 1573–1577, doi:10.1016/S0277-5387(00)84559-3.
